# A Single‐Base Mutation in *TaWAK3‐B* Reduces Plant Height via Cytoskeleton in Bread Wheat

**DOI:** 10.1111/pbi.70563

**Published:** 2026-01-29

**Authors:** Naijiao Wang, Ruolin Bian, Dejie Du, Yunjie Liu, Yiao Ma, Zihao Jiang, Zhaoju Li, Yan Zhou, Xiangyu Zhang, Zhaoheng Zhang, Beilu Cao, Xiongtao Li, Zhaoyan Chen, Jie Liu, Qixin Sun, Zhongfu Ni, Lingling Chai

**Affiliations:** ^1^ State Key Laboratory of High‐Efficiency Production of Wheat‐Maize Double Cropping, Frontiers Science Center for Molecular Design Breeding, Key Laboratory of Crop Heterosis and Utilization (MOE), Key Laboratory of Crop Genetic Improvement China Agricultural University Beijing China

**Keywords:** cytoskeleton, plant height, TaADF3‐A, TaIQD2‐D, TaKLCR1‐A, TaWAK3‐B, wheat

## Abstract

Introduction of *Reduced height* (*Rht*) genes into modern wheat cultivars has resulted in ‘Green Revolution’ that skyrocketed wheat grain yields worldwide since the 1960s. These ‘Green Revolution’ cultivars show shorter plant height, but higher lodging resistance and harvest index. The identification and exploitation of novel *Rht* genes are of great significance for the development of high‐yielding wheat cultivars. In this study, a semi‐dwarf wheat mutant, *d14078*, with reduced plant height and grain size, was generated by ethyl methanesulfonate (EMS) mutagenesis. Here, through map‐based cloning, we cloned the causal gene for the semi‐dwarf phenotype of *d14078* as *TaWAK3‐B* that encodes a cell wall‐associated receptor kinase 3. A single‐base mutation occurred in the coding region of *TaWAK3‐B*, resulting in an amino acid mutation from Glu to Lys (E938K) at residue 938, which reduces its stability and the formation of homodimers. The cytoskeletons were changed in both the *d14078* and *TaWAK3‐B* knockout mutants, as well as the *TaWAK3‐B* overexpression of transgenic plants. Further investigation revealed that TaWAK3‐B directly forms stable protein assembly with TaADF3‐A (actin depolymerisation factor), TaKLCR1‐A (kinesin light chain‐related protein 1), and TaIQD2‐D (IQ67‐domain protein 2). These interactions and complex formations were significantly attenuated by the TaWAK3‐B^E938K^ mutation. Therefore, our findings clarify *TaWAK3‐B* regulating the microfilament and microtubule formation that elucidate on the regulation of wheat stem development.

## 1 Introduction

Bread wheat ( *Triticum aestivum* L., AABBDD, 2*n* = 6*x* = 42) is one of the most important food crops in the world, providing one‐fifth of the calories for humans (Dubcovsky and Dvorak 2007). In the 1960s, the introduction of semi‐dwarf traits in cereal crops to improve lodging resistance led to a significant increase in yields, which is known as the “Green Revolution” (Hedden 2003). According to statistics, the global population will exceed 9 billion in 30 years (Tyczewska et al. 2018). Therefore, it is essential to improve wheat grain yields.

The semi‐dwarf traits in wheat were mainly introduced by the *Reduced height* (*Rht*) gene *Rht‐B1b* (formerly *Rht1*) and *Rht‐D1b* (*Rht2*) of the wheat variety “Norin10” from Japan (Peng et al. 1999). So far, 27 loci have been designated as *Rht* genes (Peng et al. 2011; Chen et al. 2015; Mo et al. 2018; Song et al. 2023; Zhang et al. 2023; Liu et al. 2024). According to the response to exogenous application of bioactive gibberellin (GA), the *Rht* genes can be classified into GA‐sensitive group and GA‐insensitive group (Worland et al. 1998; Bazhenov et al. 2015; Chen et al. 2015; Zhao et al. 2018). *Rht‐B1* and *Rht‐D1* encode DELLA proteins, which are key repressors in the gibberellic acid (GA) signalling pathway. The *Rht‐B1b* and *Rht‐D1b* mutant alleles harbour nucleotide substitutions in their DELLA motif‐coding regions, leading to premature translation restart and accumulation of truncated N‐terminal DELLA proteins. These stable proteins constitutively repress GA signalling without affecting GA catabolism, ultimately resulting in a GA‐insensitive semi‐dwarf phenotype (Peng et al. 1997, 1999; Richards et al. 2001; Bazhenov et al. 2015; Van de Velde et al. 2021). There are a series of adverse traits of *Rht‐B1b* and *Rht‐D1b*, including shorter germ stage, decreased seedling vigour, and reduced grain size, which resulted in yield loss (Richards 1992; Rebetzke et al. 1999; Ellis et al. 2004; Zhang et al. 2013; Wuerschum et al. 2017; Guan et al. 2018). Therefore, some new *Rht* loci have been used as alternative genetic resources for wheat semi‐dwarf breeding. A rare haplotype *r‐e‐z* was recently identified with *ZnF* positively regulating BR signalling and *Rht‐B1* negatively regulating GA signalling. This haplotype‐mediated BR‐GA hormonal rebalancing shows potential breeding value for breaking the yield bottleneck of existing “Green Revolution” varieties (Song et al. 2023). *Rht8* is one of the most important GA‐sensitive genes in wheat semi‐dwarf breeding, which can shorten the plant height of wheat by ~10% without affecting coleoptile elongation and grain yield (Rebetzke et al. 1999). *Rht8* is localised to the short arm of the 2D chromosome and encodes a nucleoprotein of 808 amino acids, which contains a predicted ribonuclease H‐like domain (Gasperini et al. 2012; Chai et al. 2018, 2022). *Rht18* is another GA‐sensitive semi‐dwarf locus mapped to chromosome 6A. *GA2oxA9*, encoding the ga‐inactivating enzyme GA2‐oxidase, is a candidate gene for *Rht18* (Ford et al. 2018). It has been found that *Rht18*, *Rht24*, and *Rht14* may be related to overlapping regions, and *Rht24b* not only reduces plant height but also improves nitrogen use efficiency without causing yield loss (Tian et al. 2017; Wuerschum et al. 2017; Ford et al. 2018; Mo et al. 2018). *Rht12* was found in wheat γ radiation‐mutagenic, localised on chromosome 5A, and *GA2oxA13* (also referred to as *GA2oxA14* in another report) was proved a candidate gene and functionally similar to *Rht18* (Sutka and Kovacs 1987; Ellis et al. 2004; Sun et al. 2019; Buss et al. 2020). *Rht23* is a dominant gene on chromosome 5DL that confers GA‐insensitivity, dwarfism, and compact spikes via altered cellular anatomy (Chen et al. 2015). The *Rht23* mutant exhibits dwarf and compact spike phenotypes. This locus was mapped to the chromosomal region harbouring *5Dq*, and a SNP within *5Dq* was found to co‐segregate with the mutant traits, indicating tight genetic linkage (Zhao et al. 2018). *Rht25* was identified on chromosome 6A and there was a physical interaction with *Rht1* (Zhang et al. 2023). The researchers discovered new dwarf genes *Rht26* and *Rht27* on chromosomes 3DL and 3A (Song et al. 2023; Liu et al. 2024). The actin family genes *DRG1*/*TaACT7*/*TaACTIN7‐D* regulate plant height and grain length by affecting the organisation of F‐actin (Li et al. 2023; Xie et al. 2023).

Cell wall‐associated receptor kinases (WAKs), a plant Receptor‐Like Kinases (RLKs) subfamily, feature extracellular EGF‐like domains for protein interactions, transmembrane regions, and intracellular serine/threonine kinase domains (He et al. 1996, 1999; Shiu and Bleecker 2001). Five *WAKs* and 21 *WAK‐likes* (*WAKLs*) were found in Arabidopsis, while the number of genes of the *WAKs* family in rice was 5‐fold higher than in Arabidopsis (Zhang et al. 2005). WAKs play an important role in cell elongation, and the leaf cells of antisense plants with five *WAKs* in *Arabidopsis thaliana* are smaller than those of wild type (Anderson et al. 2001; Kanneganti and Gupta 2008), and *AtWAK4* affects cell elongation and lateral root development (Lally et al. 2001). *WAK2* in *Arabidopsis thaliana* affects cell elongation by regulating the expression level of vacuolar invertase, while vacuolar invertase increases solute concentration by decomposing sucrose, increases intracellular turgor pressure, and leads to cell wall loosening (Kohorn et al. 2006). Cell expansion is regulated in a variety of ways, among which gibberellin, brassinolide, and auxin have all been shown to be involved in the regulation of cell expansion (Jones 1998; Phillips 1998; Müssig and Altmann 1999; Silverstone and Sun 2000). Cell wall defects affect plant hormone signalling pathways, in which pectin and xyloglucan stimulate brassinolide signal transduction (Wolf et al. 2012; Aryal et al. 2020). *WAKs* have been reported to regulate cell elongation by mediating a regulatory pathway from cell wall pectin to BR signalling (Yue et al. 2022). The strength and ductility of the cell wall depend on the chemical interactions between polysaccharides and the ability to rearrange rapidly at the subcellular level (Bidhendi and Geitmann 2016). It has been reported that Arabidopsis PM‐localised receptor kinase FERONIA regulates the morphogenesis of pavement cell by combining highly dmPectin (Lin et al. 2022). The plasma membrane receptors BRASSINOSTEROID INSENSITIVE 1 (BRI1) and RECEPTOR‐LIKE PROTEIN 44 (RLP44) in Arabidopsis synergistically regulate the cell wall response due to abnormally elevated mPectin levels by BRI1‐Associated Kinase 1 (BAK1), a co‐receptor of BRI1 (Wolf et al. 2012, 2014; Glöckner et al. 2022). Cellulose synthase, an important component of the cell wall, has been reported to be involved in the orientation of cortical microtubules (Fisher and Cyr 1998; Baskin et al. 1999; Kost et al. 1999). It has been reported that WAKs are the release of receptors for OGs caused by infection with injured pathogens, and that WAKs and WAKLs are important for plant defence (Brutus et al. 2010; Ferrari et al. 2013; Menna et al. 2021; Zhong et al. 2024).

It is crucial for the cytoskeletal system to maintain the morphology of the cell, withstand external stress, maintain the internal structure of the cell, and participate in many important life structures. In plants, the cortical endoplasmic reticulum (ER) network and plasma membrane (PM) are connected by the ER‐PM contact sites (EPCSs), and their structure is maintained by EPCS‐resident proteins and the cytoskeleton, and there is a strong co‐arrangement between EPCSs and the cytoskeleton (Wang et al. 2014; McFarlane et al. 2017; Lee et al. 2019). Therefore, as an important system for the composition of the cytoskeleton, microtubules and microfilaments are essential for the maintenance of the cytoskeleton. Microfilaments are composed of actin, and actin depolymerisation factor (ADF)/cofilin family proteins are a class of actin‐binding proteins that are ubiquitous in eukaryotic cells and promote actin dynamics by promoting actin polymerisation or F‐actin depolymerisation through the molar ratio of ADF/cofilin protein to actin (Bowman et al. 2000; Van Troys et al. 2008; Zhao et al. 2015; Ueda et al. 2018). Microtubules are the architectural backbone of the cytoskeleton and are composed of α and β tubulin, which are usually found as dimers. It has been found that in Arabidopsis, two microtubule‐binding proteins, kinesin light chain‐associated protein 1 (KLCR1) and IQ67‐domain protein 2 (IQD2), together with the actin‐binding protein NET3C, form a component of plant EPCS and mediate the connection between the actin and microtubule networks (Zang et al. 2021).

In this study, a semi‐dwarf wheat mutant line *d14078* produced by EMS mutagenesis was identified based on the genetic background of wheat cultivar Jinmai47. Further map‐based cloning revealed that a single‐nucleotide mutation in *TaWAK3‐B* explained the reduced plant height of *d14078*. Our further results demonstrate that *TaWAK3‐B* regulates the morphogenesis of pavement cells by regulating proteins of microfilaments and microtubules, resulting in the reduction of plant height in wheat. Interestingly, the overexpression of *TaWAK3‐B* accelerated heading date without compromising grain yield in wheat. These findings provide insight into wheat plant height development and provide important genetic resources for wheat architecture research.

## 2 Results

### 2.1 Phenotypic Characterisation of the Wheat Semi‐Dwarf Mutant *d14078*

**FIGURE 1:**
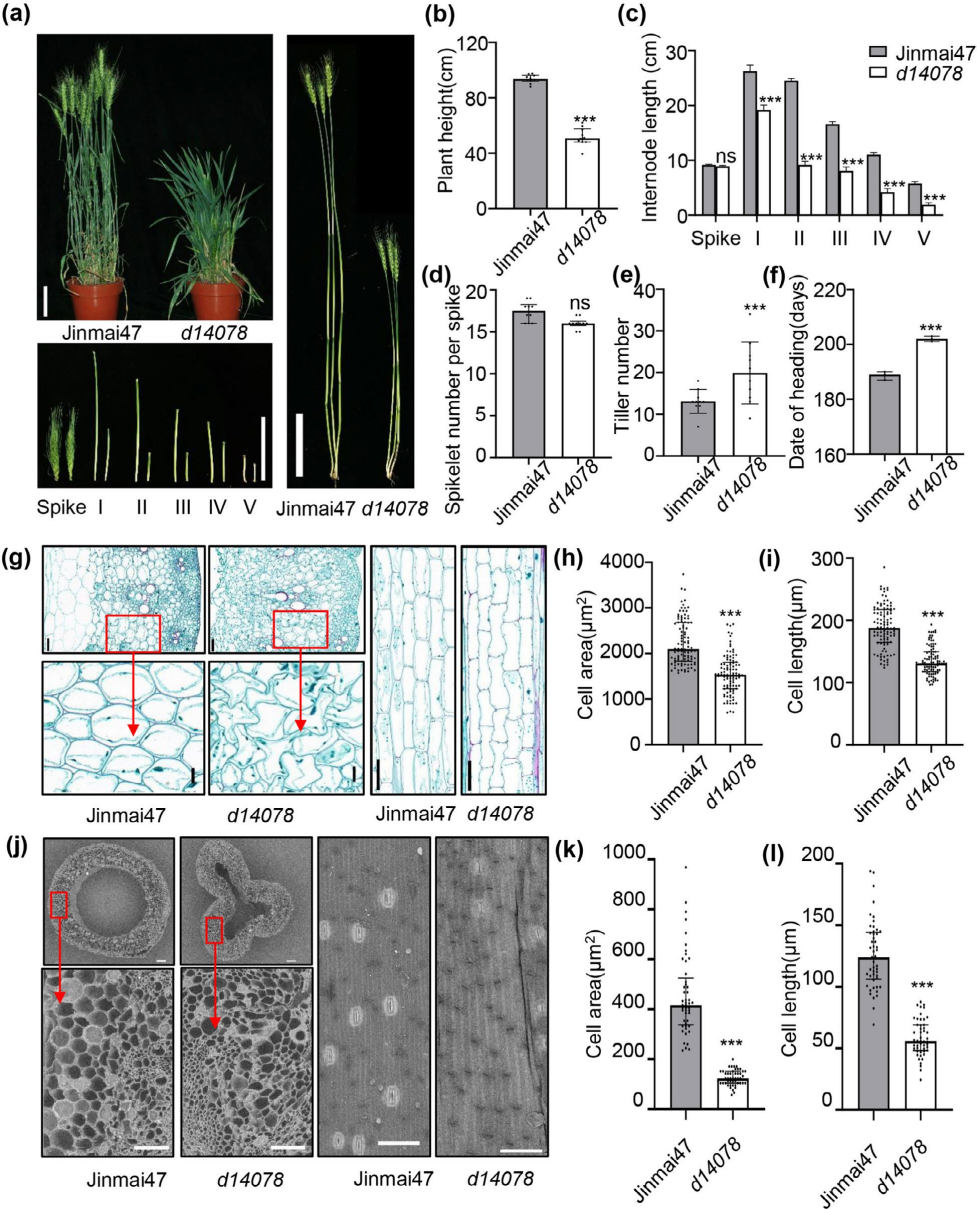
Growth phenotypes and cytological characteristics of bread wheat ( *Triticum aestivum* L.) cultivar Jinmai47 and its derived mutant line *d14078*. (a) Plant height, spike length and each internode of Jinmai47 and *d14078*, bars, 10 cm. I‐IV indicate the different stem segments of Jinmai47 and *d14078*. (b–f) Statistical analyses of plant height (b), the length of spike and each internode (c), spikelet numbers per spike (d), tiller number (e) and the date of heading (f) of Jinmai47 and *d14078*. (Plant height, internode length of stems, spikelet number per spike and tiller number, *n* = 10; date of heading, *n* = 3). (g) Cell sizes of the peduncles of Jinmai47 and *d14078*, bars, 100 μm. (h and i) Statistical analyses of the cell area (h) and cell length (i) of Jinmai47 and *d14078* (*n* = 99). (j) Scanning electron microscopy images of Jinmai47 and *d14078*, bars, 100 μm. (k and l) Statistical analyses of the cell area (k) and cell length (l) showing the peduncle pericarp of Jinmai47 and *d14078* (*n* = 49). In (g) and (j), the stems were collected at flowering stage. Data are means ± SD. SD, standard deviation. (Student's *t*‐test, ns, not significant, ***p < 0.001).

*d14078*, a dwarf mutant line, was generated through EMS mutagenesis from the genetic background of the common wheat cultivar Jinmai47. Via phenotypic analysis, the *d14078* mutant displays a semi‐dwarfing phenotype, with significantly reduced stem length, increased tiller numbers, and significantly increased growth period (Figure 1a–f). Statistical analysis confirmed that the plant height reduction in *d14078* was due to the shortening of every internode. The plant height of *d14078* (51.75 ± 6.52 cm) was decreased by 44.7% compared with Jinmai47 (93.62 ± 3.02 cm; Figure 1b). The *d14078* mutant exhibited a 51.9% increase in tiller number but a 13‐day delay in heading date compared to Jinmai47 (Figure 1e–f). No significant difference was found in spike length or spikelet number per spike between the *d14078* mutant and Jinmai47 (Figure 1c,d). In addition, compared with Jinmai47, the *d14078* mutant showed significant decreases of 36.40%, 11.10%, and 12.60% in thousand‐grain weight, grain length, and grain width, respectively (Figure S1a–d).

Comparing to the straight and smooth stem in Jinmai47, the wrinkled internode was observed in *d14078* (Figure S1e,f). Through cytological analysis on the median peduncle sections of both Jinmai47 and *d14078* at the flowering stage, we found that the longitudinal cells were significantly shorter and had a smaller area in *d14078* compared to Jinmai47 (Figure 1g–i). Similarly, the stem epidermal cell was observed using electron microscopy, where the length and area were significantly reduced, and the cells were in disordered arrangement in *d14078* compared to Jinmai47 (Figure 1j–l).

### 2.2 Map‐Based Cloning of the Causal Gene for Dwarf Phenotype of *d14078*

In order to explore the genetic basis underlying the dwarf phenotype of *d14078*, we generated an F_2_ segregating population by crossing with a wheat cultivar Jing411 and the mutant *d14078*. The *d14078* mutant exhibited a 46.8% reduction in plant height compared to Jing411 (97.27 ± 2.42 cm; Figure S2b). While its spikelet number per spike decreased by 15.9% (Figure S2e), the tiller number increased by 39.2% (Figure S2f). No significant difference was observed in spike length between *d14078* and Jing411 (Figure S2d). However, Jing411 showed higher values than *d14078* in thousand‐grain weight (23.50%), grain length (9.59%), and grain width (3.52%) (Figure S2h–j).

**FIGURE 2:**
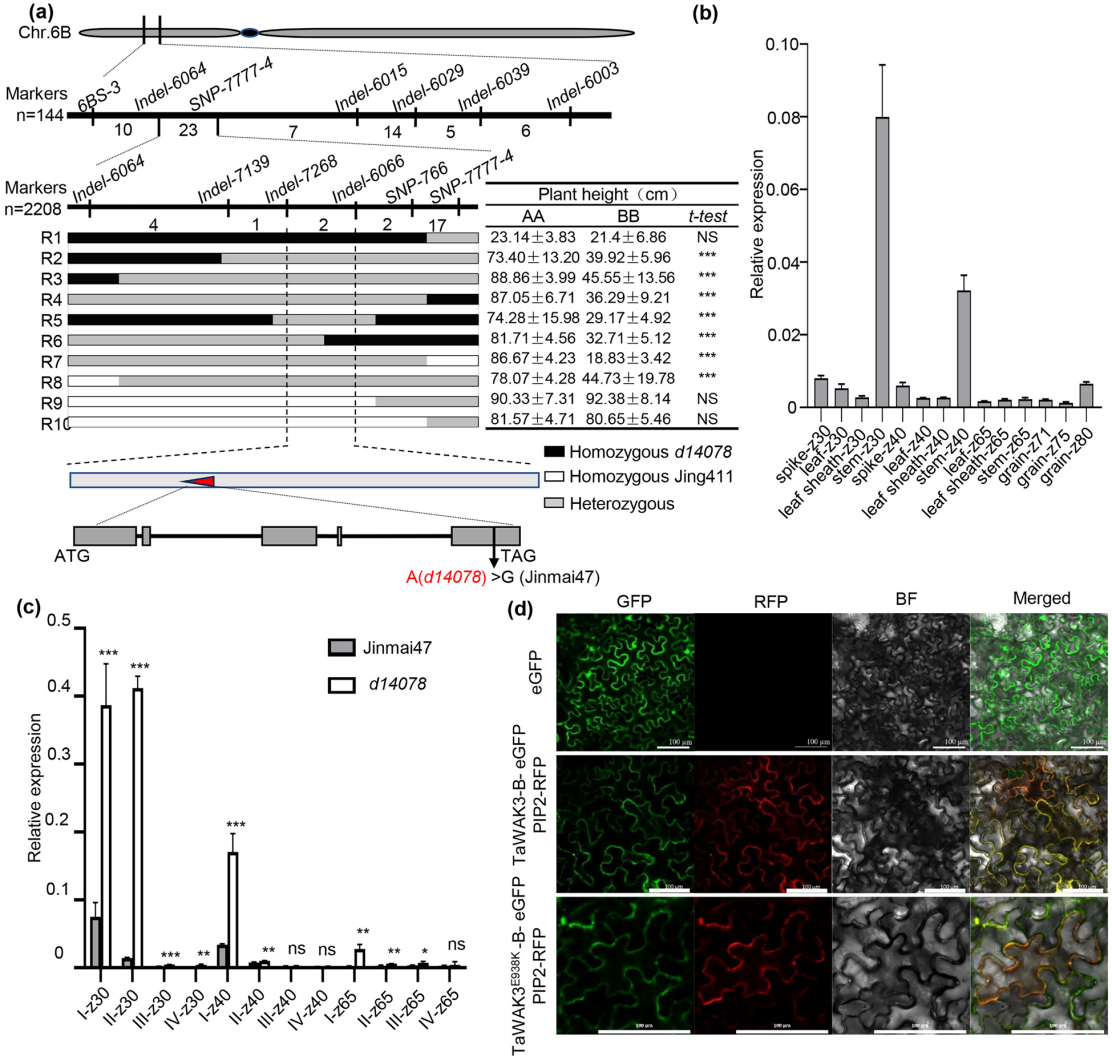
Map‐based cloning of the candidate gene *QPh.cau‐6B*. (a) Map‐based cloning of *QPh.cau‐6B* on chromosome 6B using recombinants from two F_2_ segregating population. The number of recombinants between adjacent markers is indicated, and white and black rectangles separately represent the homozygous Jing411 and *d14078* background, while grey indicates heterozygous regions. Candidate gene structure of *TraesCS6B02G097000* and its sequence variation between Jinmai47 or *d14078* were shown below. (b) RT‐qPCR analysis of *TaWAK3‐B* expression of Jinmai47 in different wheat tissues (*n* = 3). The Zadoks scale is a cereal development standard proposed by Dutch plant pathologist Jan C. Zadoks that is widely used to identify the growth stages of cereals such as wheat. Key stages include: Z30, beginning of stem elongation; Z40, beginning of booting; Z65, mid‐flowering; Z71, 1 day after pollination; Z75, 5 days after pollination; and Z80, 10 days after pollination. (c) RT‐qPCR analysis of *TaWAK3‐B* expression of Jinmai47 and *d14078* in each stem (*n* = 3). (d) Subcellular localisation of TaWAK3‐B‐GFP and TaWAK3‐B^E938K^‐GFP in *N. benthamiana* cells. Bars, 100 μm. Data are means ± SD. SD, standard deviation. (Student's *t*‐test, ns, not significant, *p < 0.05, **p < 0.01, ***p < 0.001).

We selected ten individuals that were extremely tall from the F_2_ segregating population and equally mixed their DNAs for the construction of bulk “wild‐type” DNA. Meanwhile, ten individuals with dwarf phenotypes were selected from the F_2_ segregating population to generate the bulk “mutant” DNA. BSA revealed that the single‐nucleotide polymorphisms (SNPs) with high allele frequency difference values were mainly distributed in chromosome 6B (76.29%) (Figure S3a). Thirteen SSR markers from published research and eight markers newly developed were used for genetic map construction (Figure S4a). QTL analysis with 1711 individuals from the F_2_ segregating population mapped a major QTL controlling plant height on chromosome 6B, designated as *QPh.cau‐6B* (Figures S3b,c and S4), and the additive effect of *QPh.cau‐6B* was 23.73 cm (Figure S4b). By screening the recombinants from F_2_ and F_3_ populations, *QPh.cau‐6B* was narrowed down to the interval between markers *Indel‐7268* and *Indel‐6066*, corresponding to the physical interval of the Chinese Spring (CS) reference genome (IWGSC Refseq v1.1) region of 2.88 Mb (Figure 2a). Analysis of transcriptome data using young stems at the jointing stage of Jinmai47 and *d14078* identified five genes expressed within the mapping interval, among which only *TraesCS6B02G097000* exhibited a significant expression difference between Jinmai47 and *d14078* (Figure S5). Based on sequence alignment, only *TraesCS6B02G097000* had a base substitution (“G” in Jinmai47 and “A” in *d14078*) between Jinmai47 and *d14078* (Figures 2a and S6). The G‐to‐A mutation of *d14078* results in a change from the amino acid at position 938 of the inferred protein from glutamic acid (Glu, E) to lysine (Lys, K) (Figure S7). Based on these analyses, we propose *TraesCS6B02G097000* as the candidate gene of *QPh.cau‐6B*.

Based on functional annotation, *TraesCS6B02G097000* was identified as a wheat ortholog of *AT1G21240* in *Arabidopsis thaliana* and is highly conserved across plant species (Figure S8). We therefore designated it as *TaWAK3‐B* (the B genome copy of *WAK3* in wheat) and named the mutant allele in *d14078* as *TaWAK3‐B* ^*E938K*^. *TaWAK3‐B* comprises five exons and four introns, encoding a cell wall‐associated receptor‐like kinase 3 (WAK3) protein of 1072 amino acids. Analysis of its spatiotemporal expression revealed transcript accumulation in various tissues (Figure 2b). Notably, *TaWAK3‐B* expression was significantly higher in *d14078* than in Jinmai47 (Figure 2c). To determine the subcellular localisation of TaWAK3‐B, we fused its full‐length coding sequence to GFP. The TaWAK3‐B‐GFP fusion protein localised to the cell membrane. The mutant protein TaWAK3‐B^E938K^‐GFP showed a similar localisation pattern, consistent with the known plasma membrane association of WAK/WAKL proteins in other plants (Figure 2d) (Zhong et al. 2024).

### 2.3 *TaWAK3‐B* Is the Causal Gene Controlling Plant Height in *d14078*

**FIGURE 3:**
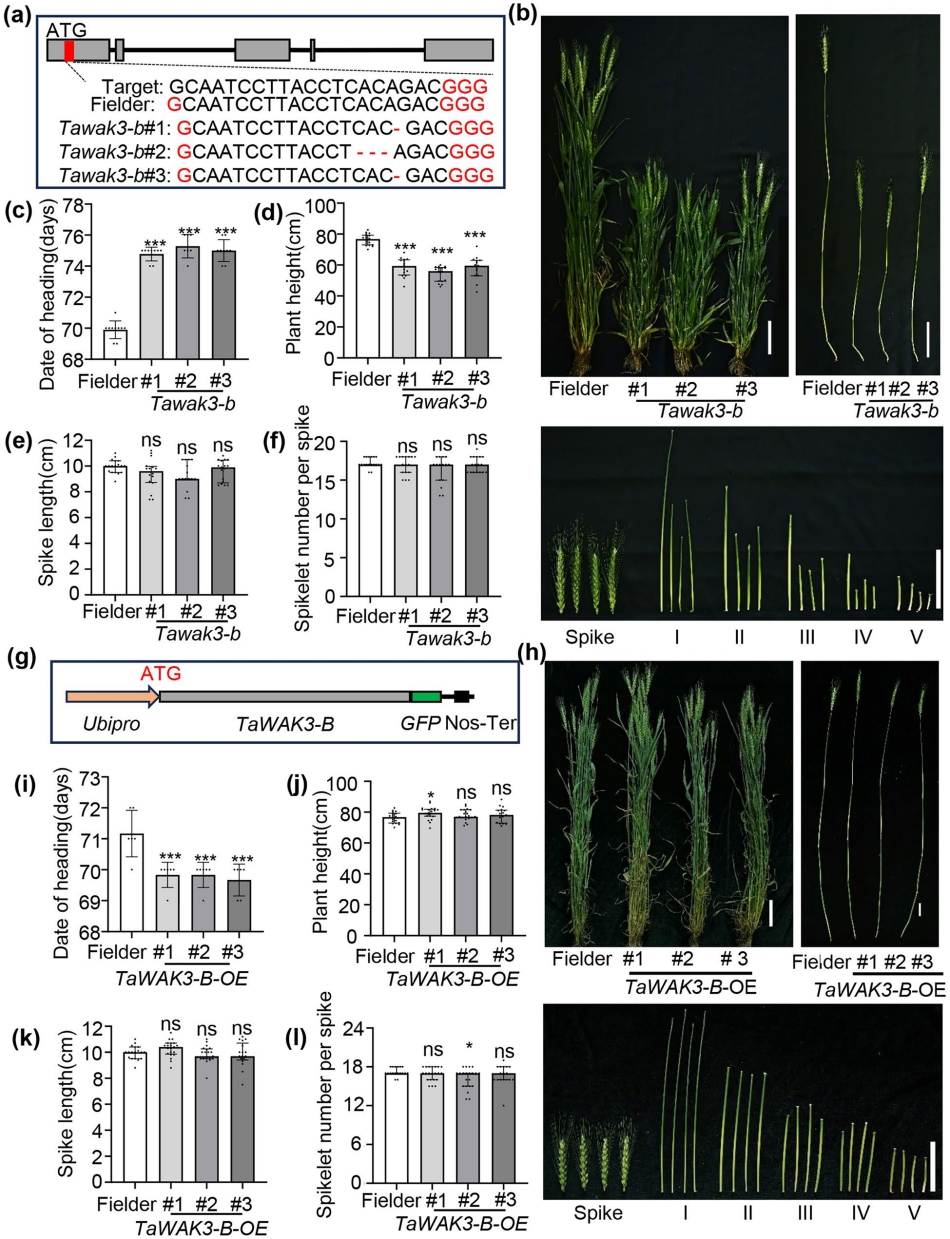
Phenotypic analyses of transgenic wheat plants in the ‘Fielder’ background. (a) CRISPR/Cas9‐mediated mutations in the *TaWAK3‐B* locus. The positions and sequences of target site for CRISPR/Cas9 gene editing are shown above. The symbols ‘–’ separately indicate the nucleotide deletion. (b) Plant height, spike length and each internode of Fielder and derivative *Tawak3‐b* mutants. Bars, 10 cm. I‐IV indicate the different stem segments of Jinmai47 and *d14078*. (c–f) Statistical analysis of date of heading of Fielder and *Tawak3‐b* mutants (*n* ^Fielder^ = 10, *n* ^*Tawak3‐b*#1^ = 9, *n* ^*Tawak3‐b*#2^ = 7 and *n* ^*Tawak3‐b*#3^ = 9). Statistical analysis of plant height (d), spike length (e) and spikelet numbers per spike (f) of Fielder and *Tawak3‐b* mutants (*n* ^Fielder^ = 18, *n* ^*Tawak3*#1^ = 16, *n* ^*Tawak3*#2^ = 15 and *n* ^*Tawak3*#3^ = 17). (g) The full‐length CDS of *TaWAK3‐B* was purified and fused with overexpression vectors which carry a maize Ubiquitin promoter and green fluorescent protein sequence (GFP). (h) Plant height, spike length and each internode of Fielder and *TaWAK3‐B‐OE* transgenic plants. Bars, 10 cm. I‐IV indicate the different stem segments of Jinmai47 and *d14078*. (i–l) Statistical analysis of date of heading of Fielder and *TaWAK3‐B‐OE* mutants (*n* = 6). Statistical analysis of plant height (j), spike length (k) and spikelet numbers per spike (l) of Fielder and *TaWAK3‐B‐OE* mutants (*n* ^Fielder^ = 18, *n* ^*TaWAK3‐B‐OE*#1^ = 21, *n* ^*TaWAK3‐B‐OE*#2^ = 21 and *n* ^*TaWAK3‐B‐OE*#3^ = 17). Data are means ± SD. SD, standard deviation. (Student's *t*‐test, ns, not significant, *, *p* < 0.05, ***, *p* < 0.001).

To validate the function of *TaWAK3‐B* in vivo, we generated knockout mutant lines in the ‘Fielder’ background by CRISPR/Cas9‐mediated gene editing. We obtained three independent *Tawak3‐b* mutant lines (#1, #2, and #3). Sequencing revealed that lines *Tawak3‐b*#1 and *Tawak3‐b*#3 harboured a single‐base deletion, while line *Tawak3‐b*#2 had a three‐base deletion (Figures 3a and S9). Consistent with the *d14078* phenotype, all *Tawak3‐b* mutant lines exhibited a similar dwarf stature (Figure 3b,d). Compared to the wild‐type ‘Fielder’, the mutants also showed a delayed heading date, although spike length and spikelet number per spike were unchanged (Figures 3c,e,f and S10). Furthermore, grain traits including thousand‐grain weight, grain length, and grain width were significantly reduced in the mutant lines (Figure S11). An interesting observation is that the complete knockout of the *TaWAK3‐B* and the overexpression in the *d14078* mutant (which encodes a functionally altered protein) result in similar phenotypes, suggesting that the mutant protein may exert a dominant‐negative effect that interferes with the function of the wild‐type protein.

In addition, we generated *TaWAK3‐B* overexpression transgenic lines in the ‘Fielder’ background by driving the expression of its full‐length coding sequence (CDS) with the maize Ubiquitin promoter (Figures 3g and S12b). Phenotypic analysis showed that TaWAK3‐B overaccumulation significantly accelerated wheat growth and development (Figures 3i and S12a). However, it did not enhance other agronomic traits, including plant height, spike length, spikelet number per spike, thousand‐grain weight, grain length, or grain width (Figures 3h,j–l and S13). These results demonstrate that high expression of wild‐type *TaWAK3‐B* and mutant *TaWAK3‐B* ^*E938K*^ produces distinct phenotypes: the former accelerates development without altering final morphology, while the latter induces dwarfism. This suggests *TaWAK3‐B* function exhibits precise dose sensitivity rather than a linear correlation with expression levels. The underlying mechanism may involve the mutant protein disrupting regulatory network homeostasis through dominant negative effects or gain of function mutations, potentially leading to constitutive activation of growth suppressing signals, thus eliciting a dwarfing phenotype absent in wild‐type overexpression.

### 2.4 Genome‐Wide Transcriptome Analysis Reveals Multiple Biological Processes Regulated by *TaWAK3‐B*

**FIGURE 4:**
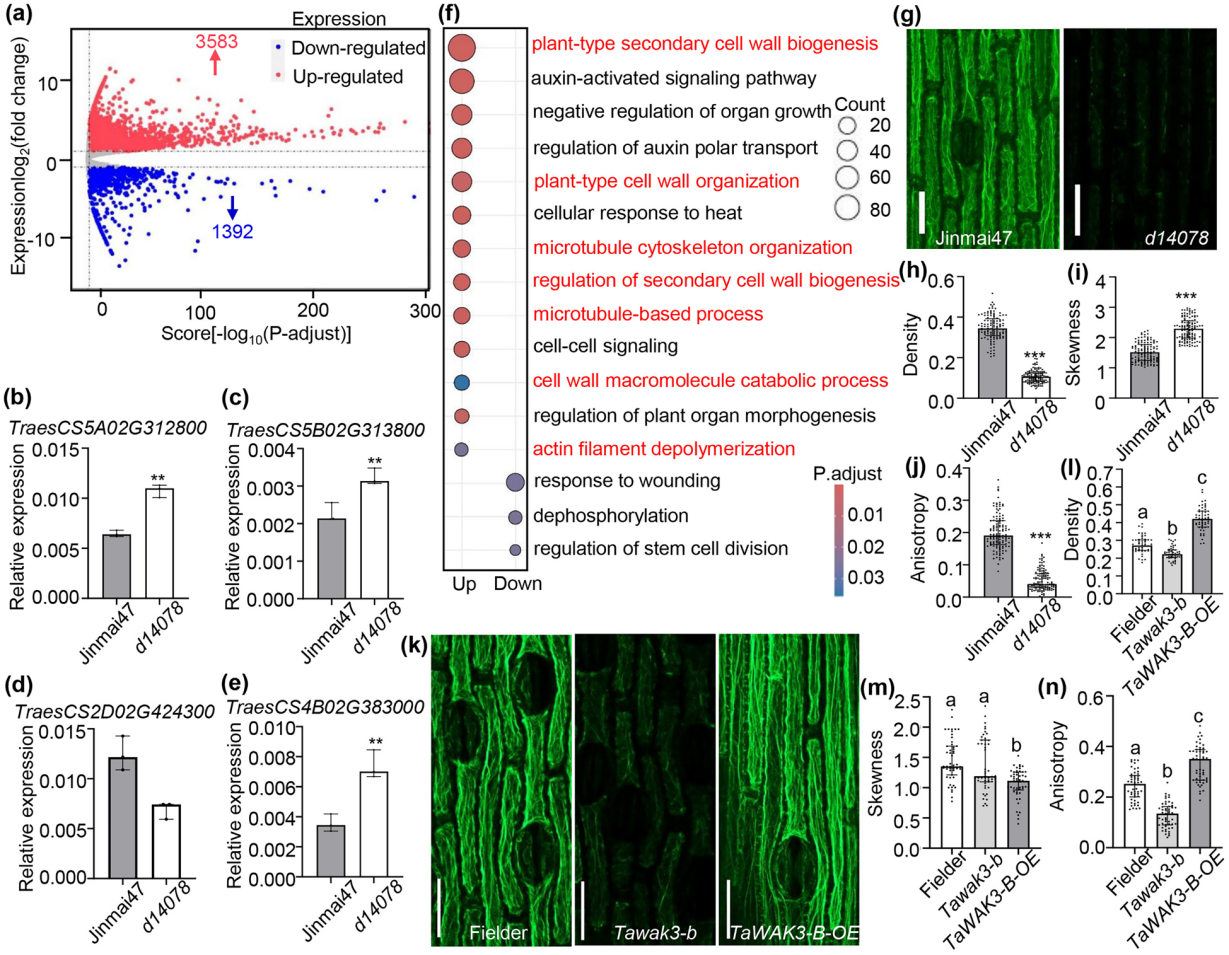
Transcriptome profiling reveals multiple signalling pathways affected by *TaWAK3‐B* ^*E938K*^ and observations of F‐Actin filament structure by fluorescent immunolabelling. (a) Volcano plot representing the DEGs in *d14078* compared to Jinmai47. The *x* axis shows the significance scores, and the y axis indicates the expression fold changes of the genes. The red and blue dots represent the upregulated and downregulated genes, respectively. (b–e) Validation of the selected DEGs include *TraesCS5A02G312800*, *TraesCS5B02G313800*, *TraesCS2D02G424300* and *TraesCS4B02G383000* by RT‐qPCR. *TraesCS5A02G312800* and *TraesCS5B02G313800* are negative regulation of organ growth genes; *TraesCS2D02G424300* is a regulation of stem cell division gene; *TraesCS4B02G383000* is a cell wall macromolecule catabolic process gene in wheat (*n* = 3). Data are means ± SD. SD, standard deviation. (Student's *t*‐test, **p < 0.01). (f) Gene ontology analysis of upregulated and downregulated DEGs shown in (A). The size of the circle indicates the number of enriched genes, and the colour scale represents the *p* value. (g) Microfilaments of the median section of peduncle at the flowering stage of Jinmai47 and *d14078*. Bars, 50 μm. (h–j) Statistical analysis of the density (h), the skewness (i) and the anisotropy (j) of Jinmai47 and *d14078* (*n* = 122). (Student's *t*‐test, ***p < 0.001). (k) Microfilaments of the median section of peduncle at the flowering stage of Fielder and *Tawak3‐b* mutant and *TaWAK3‐B‐OE* mutant. Bars, 50 μm. (l–n) Statistical analysis of the density (l), the skewness (m) and the anisotropy (n) of Fielder and *Tawak3‐b* mutant and *TaWAK3‐B‐OE* mutant (*n* = 51). The images were analysed with ImageJ software. The density, skewness, and anisotropy plugins were used to determine the density, skewness, and anisotropy of microfilaments, correspondingly [one way ANOVA, Tukey's post hoc test, *p* < 0.05].

To elucidate the signalling networks downstream of *TaWAK3‐B*, we conducted a genome‐wide transcriptome analysis using young stems of Jinmai47 and *d14078* at the jointing stage. Comparative analysis identified 4975 differentially expressed genes (DEGs) in *d14078*, with 3583 up‐regulated and 1392 down‐regulated (Figure 4a). The reliability of the RNA‐seq data was confirmed by RT‐qPCR on a subset of candidate genes, the results of which were consistent with the sequencing data (Figure 4b–e). We interrogated the expression of genes selected from transcriptome analysis in *TaWAK3‐B* knockout lines and ‘Fielder’. The consistent expression patterns in *d14078* were observed in *TaWAK3‐B* mutants (Figure S14).

Gene ontology (GO) analysis revealed that the differentially expressed genes (DEGs) were significantly enriched in multiple signalling pathways. In the *d14078* mutant, up‐regulated genes were prominently associated with cell wall‐related processes, including plant‐type secondary cell wall biogenesis, cell wall organisation, and cell wall macromolecule catabolic process. Additionally, pathways involving cytoskeletal dynamics, such as microtubule cytoskeleton organisation, microtubule‐based processes, and actin filament depolymerisation, were also significantly enriched among up‐regulated genes. Furthermore, biological processes related to plant growth and development—including negative regulation of organ growth, regulation of plant organ morphogenesis, and cellular response to heat—were significantly overrepresented in the up‐regulated genes of *d14078* (Figure 4f). In contrast, down‐regulated genes were primarily linked to response to wounding, dephosphorylation, and regulation of stem cell division (Figure 4f).

### 2.5 The E938K Mutation in TaWAK3‐B Disrupts the Normal Cytoskeletal of Stem Cell

The cytoskeleton, primarily composed of microfilaments, microtubules, and intermediate fibres (Hussey et al. 2006), plays a key role in cell structure and growth. Microfilaments are formed by the polymerisation of globular actin (G‐Actin) into filamentous actin (F‐Actin). Based on cytological and transcriptomic evidence suggesting cytoskeletal alterations in *d14078*, we examined F‐actin organisation using immunofluorescence. The skewness of the fluorescence intensity distribution, an indicator of microfilament bundling (Higaki et al. 2010), was significantly increased in *d14078* compared to Jinmai47, while F‐actin density and anisotropy were decreased (Figure 4g–j), indicating disrupted microfilament structure in *d14078*. Consistent with this, the *TaWAK3‐B* knockout lines showed reduced F‐actin density and anisotropy compared to the wild‐type ‘Fielder’, whereas the *TaWAK3‐B‐OE* overexpression lines exhibited increased density and anisotropy. Notably, skewness was significantly reduced in the overexpression lines (Figure 4k–n). These results demonstrate that *TaWAK3‐B* influences F‐actin organisation and suggest that it regulates plant height through modulation of the cytoskeleton.

### 2.6 TaWAK3‐B Physically Interacts With TaADF3‐A, TaKLCR1‐A and TaIQD2‐D

Plant EPCSs utilise cytoskeleton‐binding proteins such as NET3C, KLCR1, and IQD2 to bridge the endoplasmic reticulum and plasma membrane, facilitating actin‐microtubule interactions essential for maintaining cell structure, stress responses, and other critical functions (Zang et al. 2021). The cytoskeleton itself depends on actin filaments—regulated by ADF/cofilin—and microtubules composed of α/β‐tubulin as its core structural component (Zhao et al. 2015; Ueda et al. 2018).

**FIGURE 5:**
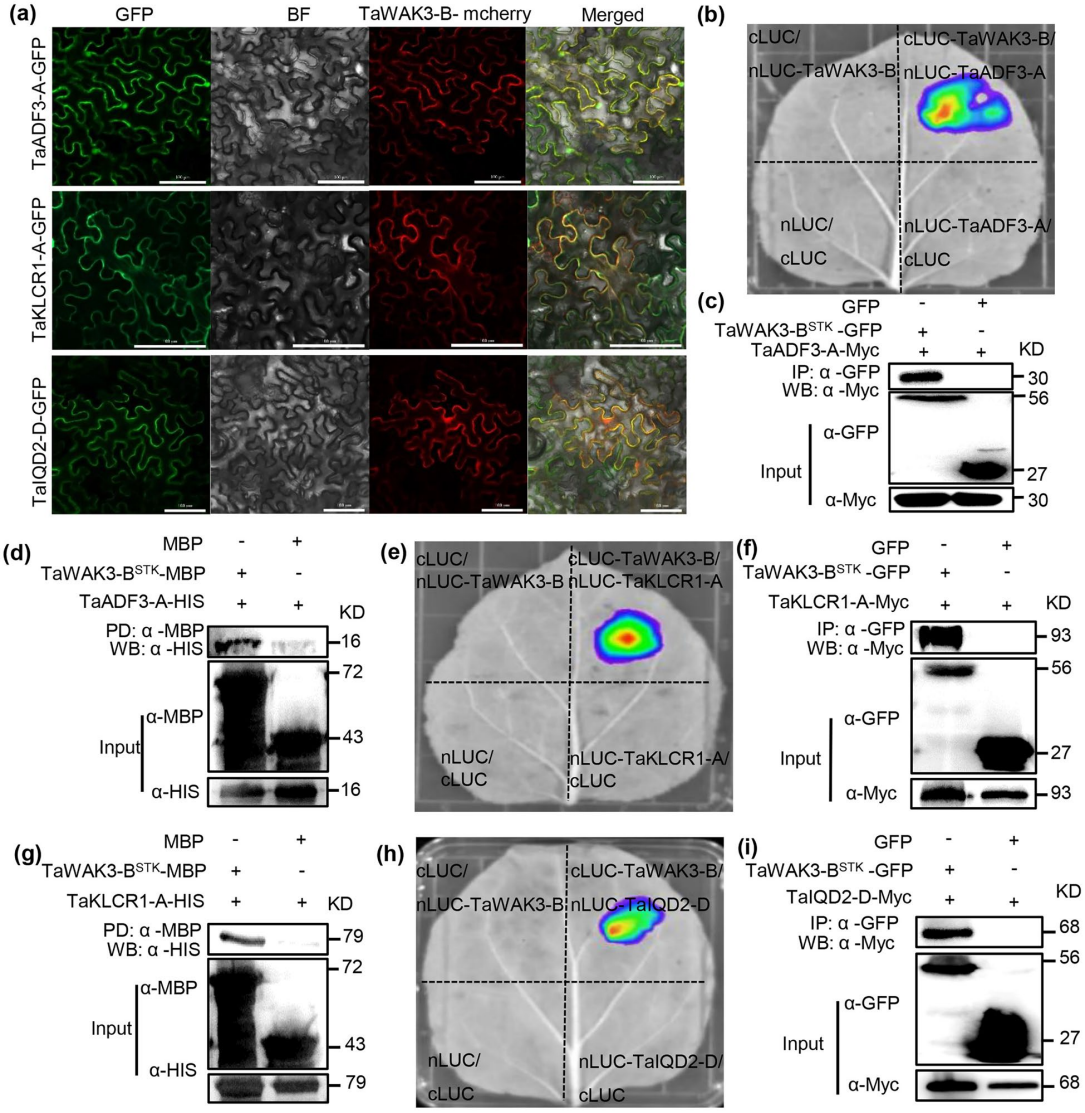
TaWAK3‐B physically interacts with TaADF3‐A with TaKLCR1‐A and TaIQD2‐D. (a) Subcellular colocalisation of TaADF3‐A‐GFP, TaKLCR1‐A‐GFP and TaIQD2‐D‐GFP with TaWAK3‐B‐mcherry in *N. benthamiana* cells. Bars, 100 μm. (b) Physical interaction of TaADF3‐A‐nLUC with TaWAK3‐B‐cLUC confirmed by the split luciferase complementation assay (SLC). (c) Physical interaction of TaADF3‐A‐Myc with TaWAK3‐B^STK^‐GFP confirmed by Co‐IP assay. (d) Physical interaction of TaADF3‐A‐HIS with TaWAK3‐B^STK^‐MBP confirmed by pull down assay. (e) Physical interaction of TaKLCR1‐A‐nLUC with TaWAK3‐B‐cLUC confirmed by the SLC assay. (f) Physical interaction of TaKLCR1‐A‐Myc with TaWAK3‐B^STK^‐GFP confirmed by Co‐IP assay. (g) Physical interaction of TaKLCR1‐A‐HIS with TaWAK3‐B^STK^‐MBP confirmed by pull down assay. (h) Physical interaction of TaIQD2‐D‐nLUC with TaWAK3‐B‐cLUC confirmed by SLC assay. (i) Physical interaction of TaIQD2‐D‐Myc with TaWAK3‐B^STK^‐GFP in Co‐IP assay.

We identified the wheat *ADF* homologue *TraesCS5A02G478900*, designated *TaADF3‐A*. Transient expression of *TaADF3‐A‐GFP* in *N. benthamiana* cells revealed its localisation to the plasma membrane and nucleus (Figure S15). TaWAK3‐B‐mcherry colocalised with TaADF3‐A‐GFP at the membrane (Figure 5a). A split‐luciferase complementation (SLC) assay confirmed a physical interaction between TaWAK3‐B‐cLUC and TaADF3‐A‐nLUC (Figure 5b). To dissect the interaction domain, we divided TaWAK3‐B into two segments: the transmembrane domain TaWAK3‐B^GUB^ and the kinase domain TaWAK3‐B^STK^ (Figure S16). The SLC assay showed stronger luminescence for the interaction of TaWAK3‐B^STK^‐cLUC with TaADF3‐A‐nLUC than with TaWAK3‐B^GUB^‐cLUC (Figure S17a,b). This interaction was further validated by co‐immunoprecipitation assay (Co‐IP), where TaADF3‐A‐Myc was coprecipitated with TaWAK3‐B^STK^‐GFP (Figure 5c), and by a pull‐down assay in which TaADF3‐A‐HIS was pulled down by TaWAK3‐B^STK^‐MBP (HIS, histidine; MBP, Maltose binding protein; Figure 5d).

Similarly, we separately cloned the homologue *KLCR1* (*TraesCS6A02G274500*, named *TaKLCR1‐A*) and *IQD2* (*TraesCS1D02G064700*, *TaIQD2‐D*) in wheat. Transient expression in *N. benthamiana* cells showed that both TaKLCR1‐A‐GFP and TaIQD2‐D‐GFP localised to the plasma membrane (Figure S15), and each colocalised with TaWAK3‐B‐mCherry (Figure 5a). SLC assays demonstrated physical interactions between TaWAK3‐B‐cLUC with TaKLCR1‐A‐nLUC or TaIQD2‐D‐nLUC (Figure 5e,h). In both cases, the kinase domain (WAK3‐B^STK^‐cLUC) mediated a stronger interaction than the galacturonan‐binding domain (TaWAK3‐B^GUB^‐cLUC) (Figure S17c–f). Co‐IP assays confirmed that both TaKLCR1‐A‐Myc and TaIQD2‐D‐Myc could be individually coprecipitated with TaWAK3‐B^STK^‐GFP (Figure 5f,i). Additionally, a pull‐down assay confirmed the direct interaction between TaKLCR1‐A‐HIS and TaWAK3‐B^STK^‐MBP (Figure 5g).

In summary, our results demonstrate that TaWAK3‐B physically interacts with TaADF3‐A (a regulator of microfilaments), TaKLCR1‐A and TaIQD2‐D (regulators of microtubules).

### 2.7 The Stability and Self‐Association of TaWAK3‐B^E938K^ Decreases and Affects the Physical Interaction With TaADF3‐A and TaKLCR1‐A

**FIGURE 6:**
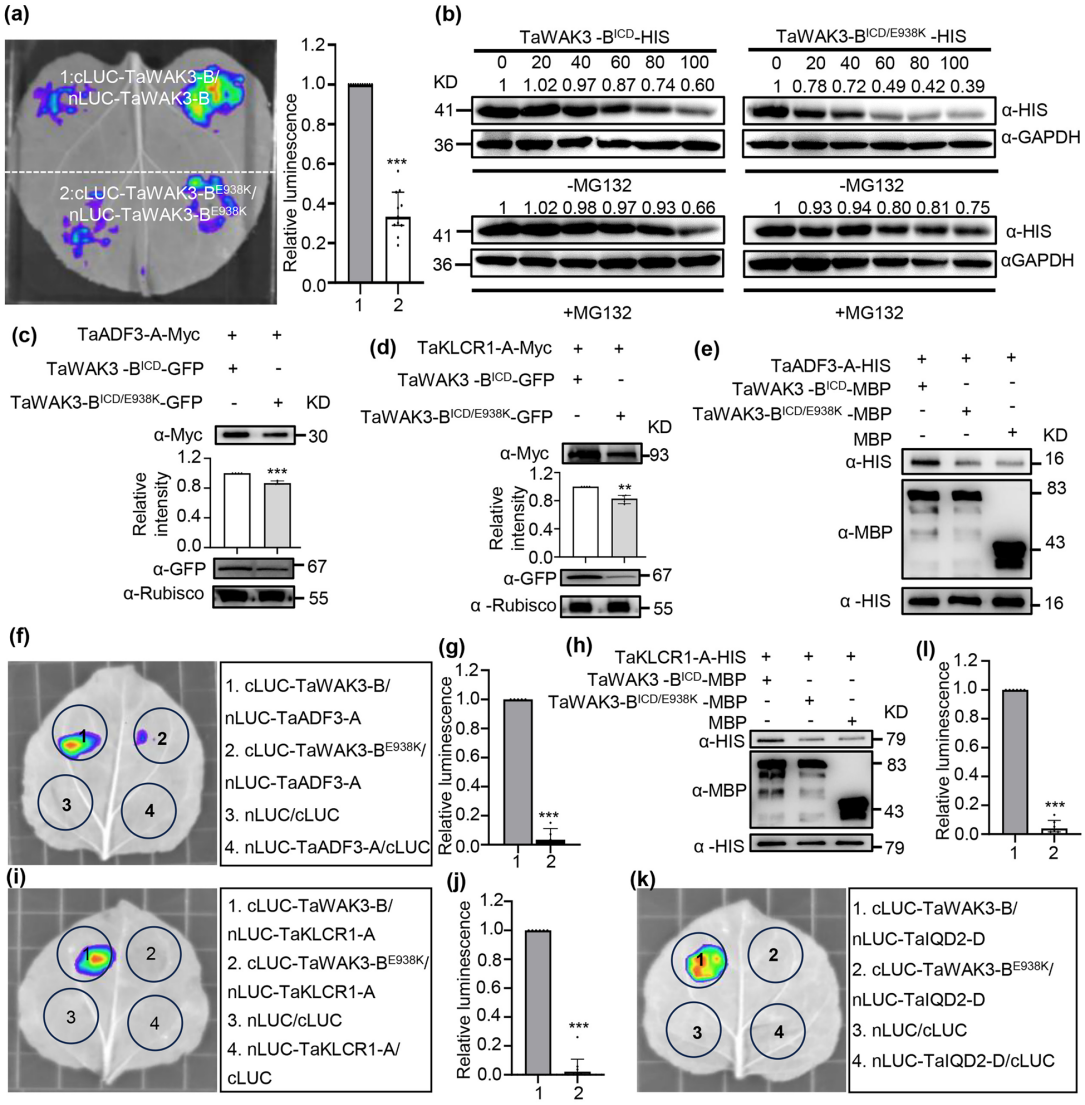
The E938K mutation affects the self‐association and stability of TaWAK3‐B protein to decrease the physical interaction with TaADF3‐A and TaKLCR1‐A. (a) Determination of TaWAK3‐B required for self‐association in a SLC assay (*n* = 11). (b) TaWAK3‐B^ICD^‐HIS was degraded faster in ‘Fielder’ cell extracts than TaWAK3‐B^ICD/E938K^‐HIS in a cell‐free degradation assay. (c) Protein complex stability assay of TaWAK3‐B^ICD^‐GFP and TaWAK3‐B^ICD/E938K^‐GFP with TaADF3‐A‐Myc in vivo (*n* = 4). (d) Protein complex stability assay of TaWAK3‐B^ICD^‐GFP or TaWAK3‐B^ICD/E938K^‐GFP with TaKLCR1‐A‐Myc in vivo (*n* = 4). (e) Physical interaction of TaADF3‐A‐HIS with TaWAK3‐B^ICD^ or TaWAK3‐B^ICD/E938K^ in a pull‐down assay. (f) Physical interaction of TaADF3‐A‐cLUC with TaWAK3‐B^ICD^‐nLUC or TaWAK3‐B^ICD/E938K^‐nLUC confirmed by a SLC assay. (g) Statistic of fluorescence value in (F), and data are means ± SD (*n* = 5). (h) Physical interaction of TaKLCR1‐A‐HIS with TaWAK3‐B^ICD^ or TaWAK3‐B^ICD/E938K^ in a pull‐down assay. (i) Physical interaction of TaKLCR1‐A‐cLUC with TaWAK3‐B^ICD^‐nLUC or TaWAK3‐B^ICD/E938K^‐nLUC confirmed by a SLC assay. (j) Statistic of fluorescence value in (I), and data are means ± SD (*n* = 6). (k) Physical interaction of TaIQD2‐D‐cLUC with TaWAK3‐B^ICD^‐nLUC or TaWAK3‐B^ICD/E938K^‐nLUC confirmed by a SLC assay. (l) Statistic of fluorescence value in (I), data are means ± SD (*n* = 6). SD, standard deviation. (Student's *t*‐test, **p < 0.01, ***p < 0.001).

Previous studies have indicated that wall‐associated kinase‐like (WAKL) proteins can form homodimers (Zhong et al. 2024), and a recent report showed that a mutant of the narrow leaf gene *TaWAK2‐A* in wheat undergoes faster degradation of protein than the wild type (Du et al. 2024). Based on these findings, we hypothesised that the ability to form homodimers may be a key factor influencing the plant height regulated by TaWAK3‐B. Wall‐associated receptor kinases typically consist of three primary components: an extracellular domain (ECD), a transmembrane domain (TMD), and an intracellular domain (ICD). Using a SLC assay, we found that TaWAK3‐B exhibits stronger homodimerisation than the mutant TaWAK3‐B^E938K^ (Figure 6a). We also assessed protein stability using a cell‐free degradation assay, which revealed that TaWAK3‐B^ICD/E938K^‐HIS was degraded more rapidly in the protein extracts of ‘Fielder’ than TaWAK3‐B^ICD^‐HIS, and this degradation was largely blocked by MG132 (Figure 6b).

We previously demonstrated that the intracellular kinase domain (STK) of TaWAK3‐B but not the extracellular galacturonan‐binding (GUB) domain interacts with TaADF3‐A, TaKLCR1‐A, and TaIQD2‐D (Figures S16 and S17). To determine whether the E938K mutation affects these interactions, we performed SLC assays and found that TaWAK3‐B^E938K^‐cLUC showed weaker interactions with TaADF3‐A‐nLUC, TaKLCR1‐A‐nLUC and TaIQD2‐D‐nLUC compared to the wild‐type protein (Figure 6f,g,i–l). Pull‐down assays confirmed that TaWAK3‐B^ICD^‐MBP pulled down a greater amount of TaADF3‐A‐HIS and TaKLCR1‐A‐HIS than TaWAK3‐B^ICD/E938K^‐MBP (Figure 6e,h). Consistent with this, in vivo protein accumulation assays showed higher levels of TaADF3‐A‐Myc and TaKLCR1‐A‐Myc when co‐expressed with TaWAK3‐B^ICD^‐GFP than TaWAK3‐B^ICD/E938K^‐GFP (Figure 6c,d).

These results suggest that the TaWAK3‐B^E938K^ variant may exhibit reduced homodimerisation capacity and an accelerated degradation rate, which further affect the binding abilities of TaADF3‐A, TaKLCR1‐A, and TaIQD2‐D, resulting in inhibition of cytoskeleton formation and semi‐dwarfing of wheat plants.

### 2.8 *TaKLCR1‐A* Regulates Wheat Plant Height

**FIGURE 7:**
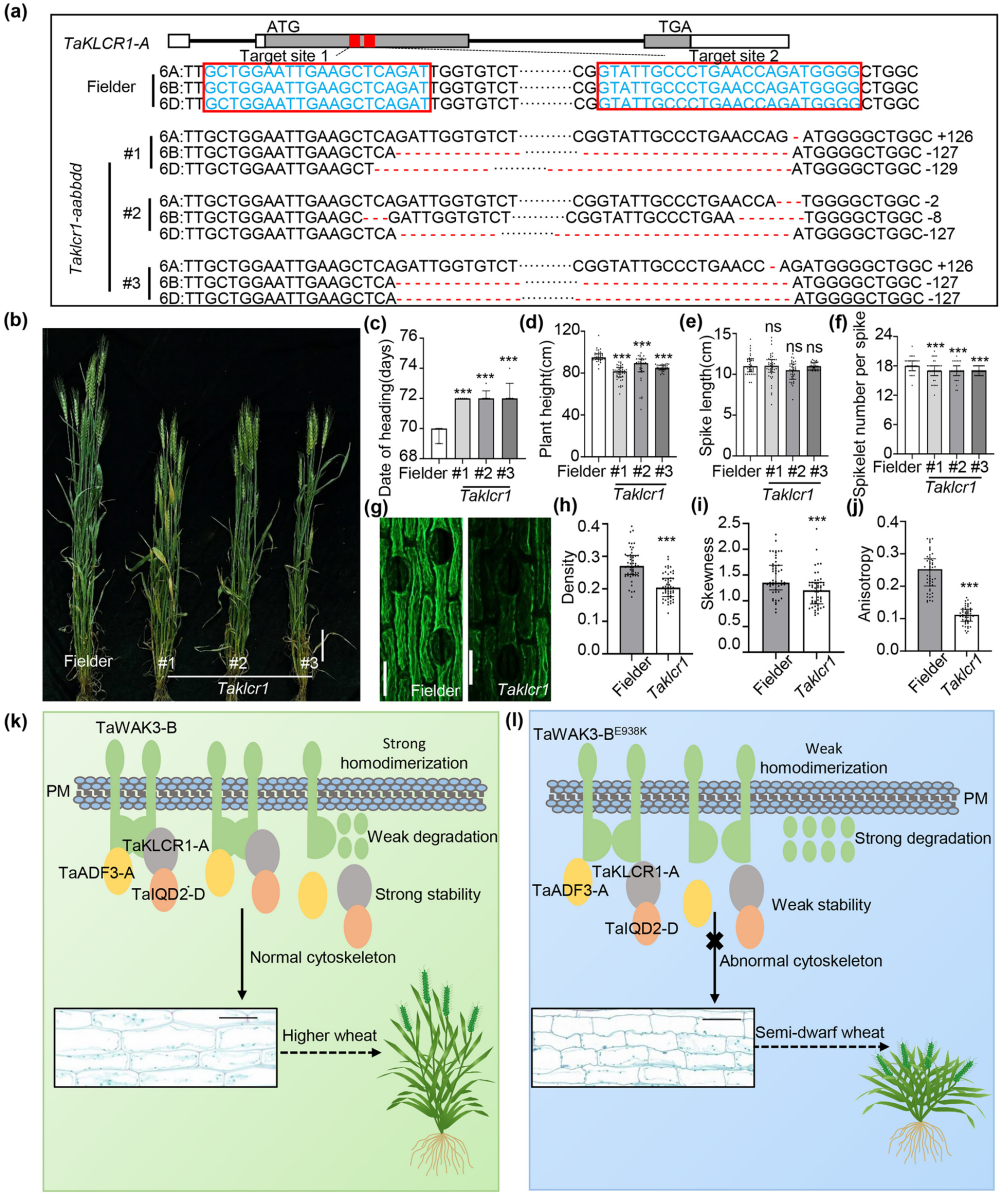
Phenotypic analyses of *TaKLCR1‐A* transgenic plants in the ‘Fielder’ background and proposed working model. (a) CRISPR/Cas9‐mediated mutations in the *TaKLCR1‐A* gene. The positions and sequences of target sites for CRISPR/Cas9 gene editing are shown above. The symbols ‘–’ separately indicate the inserted or deleted bases. (b) Plant phenotypes of ‘Fielder’ and derivative *Taklcr1* mutants. *Taklcr1#1, Taklcr1#2*, and *Taklcr1#3* are mutations on deletions or insertions in 6A/6B/6D chromosomes. Bar, 10 cm. (c) Statistical analysis of date of heading of ‘Fielder’ and *Taklcr1* mutants (*n* = 5). (d–f) Statistical analysis of plant height (d), spike length (e), and spikelet numbers per spike (f) of ‘Fielder’ and *Taklcr1* mutants (*n* ^Fielder^ = 34, *n* ^*Taklcr1*#1^ = 42, *n* ^*Taklcr1*#2^ = 33, and *n* ^*Taklcr1*#3^ = 56). (g) Microfilaments of the median section of peduncle at the flowering stage of ‘Fielder’ and *Taklcr1* mutants. The microfilament of ‘Fielder’ is the same as Figure 4k, which is the WT of these knockout mutants. Bars, 50 μm. (h–j) Statistical analysis of the density (h), the skewness (i), and the anisotropy (j) of ‘Fielder’ and *Taklcr1* mutants (*n* = 51). (k) The working model in Jinmai47 of TaWAK3‐B coordinates TaADF3‐A with TaKLCR1‐A and TaIQD2‐D to regulate the plant height in wheat. (l) The working model in *d14078* of TaWAK3‐B^E938K^ coordinates TaADF3‐A with TaKLCR1‐A and TaIQD2‐D to regulate the plant height in wheat. Data are means ± SD. SD, standard deviation. (Student's *t*‐test, ns, not significant, ***p < 0.001).

To determine whether *TaKLCR1‐A* regulates plant height, we generated *TaKLCR1‐A* knockout mutant lines in the ‘Fielder’ background using CRISPR/Cas9 technology. Due to the high conservation of TaKLCR1‐A across the wheat genome (Figure S18), we designed a strategy to simultaneously target all homologous copies located on chromosomes 6A, 6B, and 6D. Three independent mutant lines were obtained, each carrying large‐fragment deletions or insertions in the respective homologues (Figures 7a and S19). Phenotypic characterisation showed that, compared to ‘Fielder,’ the *TaKLCR1* mutant lines exhibited significantly reduced plant height, fewer spikelets per spike, lower thousand‐grain weight, and decreased grain length and width, while no significant difference was observed in spike length (Figures 7b,d–f and S21). In addition, the heading date of *the TaKLCR1* mutant lines was significantly delayed (Figures 7c and S20). To investigate whether *TaKLCR1‐A* influences microfilament organisation, we examined the F‐actin cytoskeleton in both ‘Fielder’ and *the TaKLCR1* mutant lines. Quantitative analysis revealed that the skewness, density, and anisotropy of F‐actin were significantly reduced in *the TaKLCR1* mutant lines (Figure 7g–j). Collectively, these results indicate that *TaKLCR1‐A* regulates plant height in wheat by modulating cytoskeletal morphogenesis.

## 3 Discussion

Plant height is a crucial agronomic trait that directly affects lodging resistance and ultimately grain yield. The introduction of semi‐dwarf traits during the “Green Revolution” significantly contributed to yield improvement in cereal crops (Hedden 2003). To date, more than 27 *Reduced‐height* (*Rht*) loci have been identified in wheat (Peng et al. 2011; Chen et al. 2015; Mo et al. 2018; Song et al. 2023; Zhang et al. 2023; Liu et al. 2024). Through map‐based cloning, we identified a novel dwarfing gene, *TaWAK3‐B*, and systematically elucidated its role in regulating plant height via modulation of cytoskeleton formation.

In the WAK family of proteins, single‐nucleotide substitutions can lead to loss of function. In rice, mutations in the kinase domain of *OsWAK11* disrupt signal transduction by enabling competitive binding with wild‐type proteins or forming non‐functional complexes (Yue et al. 2022). Similarly, in wheat, a single‐base mutation in *TaWAK2‐A* compromises protein stability, ultimately affecting cytokinin levels (Du et al. 2024). In the dwarf mutant *d14078*, a critical G to A nucleotide substitution occurred in *TaWAK3‐B*, resulting in the replacement of glutamic acid (E) by lysine (K) at position 938 of the encoded protein, forming the E938K missense mutation (Figures 2a and S7). This mutation site is located within a highly conserved serine/threonine kinase domain. After knocking out *TaWAK3‐B* in the ‘Fielder’ background, we observed dwarf phenotypes consistent with *d14078* (Figure 3a–f), suggesting that the E938K mutation likely disrupts the function of TaWAK3‐B. We observed that a single‐nucleotide mutation in *TaWAK3‐B* leads to decreased protein stability along with reduced self‐assembly and heteromeric assembly capacity, which may contribute to its loss of function (Figure 6a,b,e–k).

Compared to the wild‐type protein, TaWAK3‐B^E938K^ exhibits reduced self‐assembly and hetero‐assembly capacities, along with accelerated degradation (Figure 6a,b,e–k). These findings are consistent with previously reported characteristics of the WAK/WAKL protein family (Zhong et al. 2024; Du et al. 2024). We hypothesise that the E938K mutation induces conformational changes that concurrently impair interaction ability and structural stability, potentially establishing a positive feedback loop in which instability and interaction defects mutually exacerbate each other. This could lead to recognition by cellular clearance mechanisms or promote toxic aggregation, subsequently triggering degradation. These hypotheses await further experimental validation. We will continue to investigate the molecular mechanisms by which *TaWAK3‐B* regulates wheat development. Elucidating the mechanistic link between accelerated degradation and aberrant assembly will provide critical insights into *TaWAK3‐B* mediated height regulation.

We observed that compared to Jinmai47, the *d14078* mutant exhibits upregulation of intercellular signalling pathways and significantly reduced brassinosteroid sensitivity (Figure S22), suggesting that TaWAK3‐B^E938K^ may disrupt intercellular signal transduction through cytoskeletal modifications. The absence of phenotypic changes in overexpression lines shows functional parallels with the brassinosteroid signalling component BZR1: while the gain of function mutant *bzr1‐d* displays enhanced protein stability leading to dominant brassinosteroid insensitive dwarfism, overexpression of wild‐type *BZR1* produces much weaker or negligible phenotypic effects due to stringent phosphorylation‐mediated degradation control (Wang et al. 2002). Similarly, this phenomenon observed with *TaWAK3‐B* may reflect compensatory mechanisms such as dosage dependent proteotoxicity, nonfunctional hetero‐oligomerisation, depletion of upstream activators, or autoregulatory feedback. Future investigations will employ knockout and overexpression materials integrated with cytological, hormonal, and multi‐omics analyses to delineate downstream pathways, while also exploring post‐translational regulation, identifying negative regulators, and evaluating allelic variations for breeding applications.

In summary, we propose a working model in which *TaWAK3‐B* regulates plant height in wheat by modulating cytoskeleton morphogenesis (Figure 7k,l). In wild‐type plants, TaWAK3‐B forms homodimers and interacts with cytoskeletal regulators—including actin depolymerising factor TaADF3‐A, kinesin‐light‐chain‐related protein TaKLCR1‐A, and IQ67‐domain protein TaIQD2‐D—to form protein complexes that promote cytoskeleton formation and stem elongation (Figure 7k). In the *d14078* mutant, the E938K substitution reduces TaWAK3‐B stability, impairs homodimerisation, and weakens interactions with TaADF3‐A, TaKLCR1‐A, and TaIQD2‐D, thereby disrupting cytoskeleton formation and resulting in reduced plant height (Figure 7l).

## 4 Materials and Methods

### 4.1 Plant Materials and Growth Conditions

A dwarf mutant was obtained by EMS mutagenesis of Jinmai47, and the F_2_ population was obtained by crossing *d14078* with the bread wheat variety Jing411, and the phenotypic analysis (Figures S1 and S2) and map cloning of F_2_ (Figures S3 and S4) and its offspring were carried out. Jinmai47 was used as a control in this study for phenotypic observation and mechanistic studies. Wheat plants were planted in the experimental field under normal water and fertilisation conditions in Shangzhaung, Beijing, China. Wheat is sown with 20 seeds per row; each line is 1.5 m long and 0.3 m apart.

To knock out *TaWAK3‐B* and *TaKLCR1‐A*, the sgRNA target sequence was designed according to the exon sequence of these genes (http://crispr.hzau.edu.cn/CRISPR2/), and the MT1T2 vector was amplified with one or two pairs of primers containing sgRNAs and then cloned into the *pBUE411* vector. *Agrobacterium*‐mediated gene transformation technology was used to transfer the recombinant plasmid into the bread wheat variety “Fielder” (Kumar et al. 2019). The genetically encoding DNA sequences (CDSs) of *TaWAK3‐B* were amplified with TksGflex625 DNA polymerase (R060A, Takara, Japan), and the genome fragment containing *TaWAK3‐B* full‐length coding sequences was introduced into the pWMB110 vector to obtain the overexpression vector. The *agrobacterium* transformation described above was used to transfer the overexpression vector into the bread wheat variety “Fielder”. DNA from the T_0_ generation was used to verify the gene transformation. T_1_ generation leaves were used to detect the presence of transgenes by DNA and RNA to verify the expression of the gene. Plant height, spike length, spikelet number per spike, and grain traits were analysed for each transgenic line. In the transgenic experiment, ‘Fielder’ was used as a control. The WT and transgenic plants were sown with 20 seeds per row, each line is 1.5 m long and 0.3 m apart in the experimental field under normal water and fertilisation conditions in Shangzhaung, Beijing, China.

### 4.2 Phenotypic Evaluation in Field Conditions

We first classified the genotypes of individual plants, A with Jing411, B with *d14078*, and H with Jing411 and *d14078*, and then measured the plant height, spikelet number per spike, and main spike length of these plants. Independent wheat plants were harvested, and the thousand grain weight, grain length and grain width were evaluated by automatic seed particle size analysis system (SC‐G, Wanshen, Hangzhou), and the replicates were statistically analysed.

### 4.3 Cell Size Determination

Refer to previous studies to determine cell size (Chai et al. 2018). The middle section of the peduncle of Jinmai47 and *d14078* was collected and fixed in formalin‐acetic acid‐alcohol solution (FAA; 50% (v/v) ethanol, 5% (v/v) glacial acetic acid, and 4% (v/v) formaldehyde) at 4°C overnight, dehydrated in graded ethanol, and stained in xylene solution. After that, the glumes were embedded with paraffin and the tissue sections were cut into 9 μm thick sections fixed on glass slides and stained with 1% safranine and 0.5% fast green (https://www.servicebio.cn/). The obtained sections are analysed in a microscope imaging system (DS‐U3, Nikon, Japan). The images of scanning electron microscope were analysed by C.V2.4 (https://www.servicebio.cn/goodsdetail?id=1528).

The middle part of the peduncle tissue of Jinmai47 and *d14078* at the flowering stage was taken and imaged with a TM‐4000 scanning electron microscope (Hitachi, Japan). The stems of Jinmai47 and *d14078* were placed crease‐down onto aluminium specimen stubs and sputter coated with gold. These samples were observed and imaged under a Hitachi S‐3400 scanning electron microscope (Hitachi, Japan), operating at 5 kV (Wu et al. 2016; Cheng et al. 2020). Measure the length of the cells with ImageJ software (https://imagej.net/software/fiji/).

### 4.4 Map‐Based Cloning

BSA‐based map‐based cloning involves the initial step of constructing bulk DNA pools from individuals with extreme phenotypes. Through whole‐genome differential analysis, such as association mapping based on SNP‐index, the target region is preliminarily localised. Subsequently, molecular markers are developed within this region to screen recombinant individuals. By integrating genotype and phenotype co‐analysis, the candidate interval is progressively refined. Finally, the target gene is identified through functional genomics approaches. To fine map the *Qph.cau‐6B*, we performed BSA analysis using ten extremely tall plants and ten extremely dwarf plants from F_2_ segregating population of Jing411/*d14078* (Figure S3a). 1711 plants formed F_2_ segregating population of Jing411/*d14078*, and thirteen published public molecular markers and eight markers newly performed were used for initial fine mapping (Figures S3b and S4). Further fine mapping with 2208 plants composed F_2_ segregating population of Jing411/*d14078*, and newly developed markers refined the *Qph.cau‐6B* to 2.88 Mb interval between *Indel‐7268* and *Indel‐6066*. On the basis of transcriptome data and Sanger sequencing, we performed expression and sequence analysis of candidate genes in the 2.88 Mb interval and then (Figure S5), we identified the candidate gene.

### 4.5 Total RNA Extraction and RT‐qPCR

In order to analyse the expression different times and organisations of *TaWAK3‐B* in Jinmai47 and *d14078*, six leaves, six leaf sheaths, six stems and six spikes at jointing (z30), heading (z40) and flowering stages (z65), and six grains at 5 days (z71), 10 days (z75) and 15 days (z80) after flowering were taken, respectively. A total of three replicates were assessed. In order to detect the gene expression in overexpressed transgenic plants, six leaves were collected from the third leaf of the three‐leaf stage seedlings, and a total of three replicates were collected. Total RNA was extracted according to the manufacturer's protocol (Invitrogen), residual genome was removed with 4 × genomic DNA (gDNA) Wiper, and the first‐strand complementary DNA was synthesised with 1 μg of total RNA using a reverse transcription kit (Vazyme Biotech, R223‐01). RT‐qPCR assay was performed using SYBR Green PCR Master Mix (Vazyme Biotech, Q121‐02) and Real‐Time System (CFX96, Bio‐Rad). Each sample consisted of three biological replicates, and the internal control was *TaActin*.

### 4.6 RNA‐Seq and Data Analysis

The stem tissues of Jinmai47/*d14078* in jointing (z30) stage were collected for RNA extraction and library reagents, and three biological replicates were performed. Poly‐A Purification TruSeq library reagents were used for Barcoded cDNA libraries constructing, and the NovaSeq platform was used for sequencing (Illumina, San Diego, CA, USA). After screening and trimming, clean reads were mapped into the wheat reference genome (IWGSC RefSeq v1.1) using TOPHAT2 software. DEGs were used by the bioconductor package DESEQ2 (log2 (fold changes) ≥ 1 and a P value of < 0.05). GO term enrichment was determined by *Triticeae* Gene Tribe (Chen et al. 2020) (http://wheat.cau.edu.cn/TGT_beta/).

### 4.7 Gene Isolation and Sequence Analysis

To validate the CDS of *TaWAK3‐B*, *TaWAK3‐B* ^*E938K*^, *TaADF3‐A*, *TaKLCR1‐A* and *TaIQD2‐D*, these full length coding DNA sequences (CDSs) were designed for PCR detection using high‐fidelity Tks Gflex polymerase (Takara, Japan) at 94°C for 3 min, and the period is 10 s at 98°C, 15 s at 58°C, and 90 s at 68°C for a total of 34 cycles, then 68°C for 5 min. PCR products were detected on a 1% agarose gel stained with ethidium bromide and observed under UV light. Twenty bases of ATG backwards and twenty bases of TGA forward which were reversed to complement each other of these genes as amplification primers. The purified PCR products were cloned onto the pEASY‐Blunt vector (Trans Gen, China) and sequenced, after which the sequencing results were analysed for nucleotide and amino acid sequences using DNAMAN8 software and the online tool Conserved Domain Search (https://www.ncbi.nlm.nih.gov/).

### 4.8 Protein Subcellular Localisation Assays

The full length CDSs of *TaWAK3‐B*, *TaWAK3‐B* ^*E938K*^, *TaADF3‐A*, *TaKLCR1‐A*, and *TaIQD2‐D* were amplified and fused with *pCAMBIA1300‐GFP* vector to generate *TaWAK3‐B‐GFP*, *TaWAK3‐B* ^*E938K*^ *‐GFP*, *TaADF3‐A‐GFP, TaKLCR1‐A‐GFP*, *and TaIQD2‐D‐GFP*. These vectors were co‐expressed with *PIP2‐RFP* that was used for a membrane marker, which were expressed in *N. benthamiana* epidermal leaf cells via *Agrobacterium tumefaciens* (GV3101) mediated transient transformation, and after 48 h of infiltration, they were fused with a confocal microscope and detected fluorescence signal at 488 and 546 nm (LSM488; Carl Zeiss, Heidenheim, Germany).

### 4.9 Fluorescence Labeling of F‐Actin

To study the cytoskeleton, we observed F‐actin for Jinmai47, *d14078*, ‘Fielder’, TaWAK3‐b and *TaWAK3‐B‐OE*. Fresh samples were fixed in 50 mM PIPES buffer (pH = 6.9) with 4% paraformaldehyde for 3 h. After three washes in 50 mM PIPES buffer, samples were incubated in 20 nM iFluor 488 Phalloidin diluted in 50 mM PIPES buffer for 3 h, and then were incubated in a confocal microscope and detected fluorescence signal at 488 nm (LSM488; Carl Zeiss, Heidenheim, Germany), and the resulting images were analysed for skewness, density and anisotropy using ImageJ software.

### 4.10 Luciferase Complementation Imaging (LCI) Assay

For the LCI assays, the full length and truncated CDSs of *TaWAK3‐B* and *TaWAK3‐B* ^*E938K*^, and the full length of *TaADF3‐A*, *TaKLCR1‐A* and *TaIQD2‐D* were separately fused with the *pCAMBIA1300‐cLUC* and *pCAMBIA1300‐nLUC* vectors. The recombinant plasmids were transferred into *A. tumefaciens* (strain GV3101) and combined according to the experimental purpose to co‐penetrate into the leaves of *N. benthamiana*. The infiltrated leaves were exposed to light for 24 h in the dark for 24 h, and after applying 1 mM d‐luciferin (Coolaber, Beijing, China), the interaction signal was detected with the NightSHADE LB985 (Berthold Technologies, Bad Wildbad, Germany) system. Six independent leaves were analysed per experiment, and three replicates were performed per experiment. The results were similar.

### 4.11 Co‐Immunoprecipitation (Co‐IP) Assay

For Co‐IP assays, the CDSs of *TaWAK3‐B* ^*STK*^ (Figure S16) were cloned into *pCAMBIA1300‐GFP* vector to generate *TaWAK3‐B* ^*STK*^ *‐GFP* expression vector, and full‐length CDSs of *TaADF3‐A*, *TaKLCR1‐A* and *TaIQD2‐D* were cloned into *pCAMBIA1300‐Myc* vector to generate *TaADF3‐A‐Myc*, *TaKLCR1‐A‐Myc* and *TaIQD2‐D‐Myc* expression vectors. The recombinant plasmids were transferred into *A. tumefaciens* (strain GV3101), and then different expression vectors were co‐infiltrated into the leaves of *N. benthamiana* in the specified combination, which was detected with Co‐IP after 24 h of darkness and 24 h of light.

Total proteins were extracted with lysis buffer (50 mM Tris–HCl pH 7.5, 150 mM NaCl, 5 mM EDTA pH 8.0, 0.1% Triton X‐100, 0.2% NP‐40, 0.6 mM PMSF, 1× protease inhibitor cocktail, and 10 μM MG132), and then the lysates were successively incubated at 4°C for 30 min and 13 000 g for 20 min. The supernatants were incubated in 20 μL of anti‐GFP‐conjugated magnetic agarose beads (HT801; TransGen Biotech, Beijing, China) for 3 h at 4°C and then washed four times with wash buffer (50 mM Tris–HCl pH 7.5, 150 mM NaCl, 5 mM EDTA pH 8.0, 0.6 mM PMSF, and 1 × protease inhibitor cocktail). In the western blotting assay, 10% SDS‐PAGE separation was used, and anti‐GFP (mouse; 1:2000 dilution; HT801; TransGen Biotech, Beijing, China) and anti‐Myc (mouse; 1:2000 dilution; HT101; TransGen Biotech) for GFP‐tagged proteins and Myc‐tagged proteins detections, respectively.

### 4.12 In Vitro Pull‐Down Assay

For pull‐down assay, the CDSs of *TaWAK3‐B* ^*STK*^, *TaWAK3‐B* ^*ICD*^, *TaWAK3‐B* ^*STK/E938K*^ and *TaWAK3‐B* ^*ICD/E938K*^ were introduced into *pMAL‐C2x*, while *TaADF3‐A* and *TaKLCR1‐A* were fused into *pET‐32a*. Protein expression, purification, and pull‐down assays were performed on previous studies with minor modifications (Kim and Hakoshima 2019). 1 μg of the MBP or MBP‐fusion proteins and HIS‐fusion proteins were incubated with MBP beads in 1 mL of binding buffer [2.5 mM Tris–HCl (pH 7.5), 5 mM NaCl and 2 mM DTT] at 4°C for 3 h. After collecting the mixture, centrifuge at 500 g for 5 min and wash six times with the same buffer. The immunoprecipitated proteins were isolated with 10% SDS‐PAGE, and anti‐MBP antibody (mouse; 1:2000 dilution; TransGen Biotech, HT701) and antibodies anti‐His (mouse; 1:2000 dilution; TransGen Biotech, HT501) for MBP‐tagged proteins and HIS‐tagged proteins detections, respectively.

### 4.13 Cell‐Free Protein Degradation Assays

For cell‐free protein degradation assay, we extracted total protein from 2‐week‐old seedlings of “Fielder” used extraction buffer [50 mM Tris‐MES (pH 8.0), 0.5 M sucrose, 1 mM MgCl2, 10 mM EDTA (pH 8.0), 5 mM dithiothreitol (DTT), 1 mM PMSF and 1× protease inhibitor cocktail]. Because TaWAK3‐B is a membrane protein, it is difficult to purify proteins from bacteria. Therefore, we purified the ICD of *TaWAK3‐B* and *TaWAK3‐B* ^*E938K*^ for further analysis. Approximately 2 μg of HIS‐TaWAK3‐B^ICD^ and HIS‐TaWAK3‐B^ICD/E938K^ proteins were incubated separately with equal amounts of total protein from Fielder and 10 mM adenosine 5′‐triphosphate (ATP) with or without 50 μM MG132 at 25°C in the indicated times. The amounts of HIS‐tagged proteins were determined with 10% SDS‐PAGE using anti‐HIS (mouse; 1:2000 dilution; TransGen Biotech, HT501) antibody.

### 4.14 In Vivo Protein Degradation Assay

For in vivo protein degradation assay, the ICD of *TaWAK3‐B* and *TaWAK3‐B* ^*E938K*^ fused with *pCAMBIA1300‐GFP* vector and the full length CDSs of *TaADF3‐A* and *TaKLCR1‐A* fused with *pCAMBIA1300‐Myc* vector to generate *TaWAK3‐B* ^*ICD*^ *‐GFP*, *TaWAK3‐B* ^*ICD/E938K*^ *‐GFP*, *TaADF3‐A‐Myc* and *TaKLCR1‐A‐Myc* expression vectors. These vectors were transformed into *A. tumefaciens* (strain GV3101), *TaWAK3‐B* ^*ICD*^ *‐GFP* or *TaWAK3‐B* ^*ICD/E938K*^ *‐GFP* with *TaADF3‐A‐Myc* or *TaKLCR1‐A‐Myc* co‐expressed separately in leaf of *N. benthamiana*, collecting equal amounts of these leaves after 24 h in darkness and 24 h in light. The GFP‐tagged proteins were detected using anti‐GFP (mouse; 1:2000 dilution; HT801; TransGen Biotech, Beijing, China) and the Myc‐tagged proteins were detected using anti‐Myc (mouse; 1:2000 dilution; HT101; TransGen Biotech).

### 4.15 BR Sensitivity Analysis

BR sensitivity was evaluated by an LJI assay in response to eBL treatment (Tian et al. 2021). Excised 1‐cm‐long segments containing lamina joints of second leaves from 2‐week seedlings and placed them in different concentrations of 24‐epiBL solution and incubated for 48 h in the dark. Measure the lamina joint angles with ImageJ (https://imagej.net/software/fiji/) software. All experiments were repeated 3 times.

### 4.16 Statistical Analysis

Plant height was measured with a metric tape ruler. A two Student's *t* test was used for assessing the differences between genotypes or treatments. The Shapiro–Wilk test was used to check the normality when the data were more than two genotypes. If the data were normally distributed, the one‐way analysis of variance (ANOVA) followed by Tukey's post hoc test was conducted for statistical analyses. The Kruskal‐Wallis test and Dunnett's were used to compare genotypes or treatments when the normality assumption was not met.

## Author Contributions

L.C. and Z.N. conceived the project; N.W. and R.B. performed the map‐based cloning; N.W., D.D., Y.M., Z.J., Z.L., Y.Z., X.Z., B.C., and X.L. performed the experiments; Y.L. and Z.Z. analysed the sequencing data; L.C., Z.N., J.L., and Z.C. analysed experimental data; N.W., L.C., and Z.N. wrote the article; Q.S. provided critical editing of the manuscript.

## Funding

This work was supported by National Key Research and Development Program of China (Grant 2023YFD1202904), National Natural Science Foundation of China (Grants 32101767 and 32001539), Chinese Universities Scientific Fund (Grant 2025TC135), Pinduoduo‐China Agricultural University Research Fund (Grant PC2023A01003), and Bayannaoer Research Institute Young Scientist Project (Grant 2024BYNECAU006).

## Conflicts of Interest

The authors declare no conflicts of interest.

## Supporting information


**Table S1:** Primers and sequences used in this study.

**Table S2:** Enrichment analysis of single‐nucleotide polymorphisms (SNPs) based Bulked Segregant Analysis (BSA) of Jinmai47 and *d14078*.

**Table S3:** QTL analysis of plant height in Jing411/*d14078* F_2_ segregation populations.

**Table S4:** Detailed information of five annotated high‐confident genes in the 2.88‐Mb region from transcriptome data.

**Table S5:** Differentially expressed genes (DEGs) identified in stems of Jinmai47 and *d14078*.

**Table S6:** Gene ontology (GO) term enrichments of DEGs in *d14078* mutant.

**Figure S1:** Grain traits of Jinmai47 and *d14078*.
**Figure S2:** Plant phenotypes and grain traits of Jing411, *d14078* and Jinmai47.
**Figure S3:** A major QTL Controlling plant height on 6B chromosome.
**Figure S4:** Genetic linkage map and QTL analysis of *QPh.cau‐6B*.
**Figure S5:** Transcription levels of candidate genes in the *QPh.cau‐6B* mapping interval by RNA‐seq.
**Figure S6:** Nucleotide sequence alignment of *TaWAK3‐B* gene from Jinmai47 and *d14078*.
**Figure S7:** Amino acid sequence alignment of TaWAK3‐B from Jinmai47 and *d14078*.
**Figure S8:** Phylogenetic analysis of TaWAK3‐B sequences from different species.
**Figure S9:** Amino acid sequence alignment of CRISPR/Cas9‐mediated mutations in the *TaWAK3‐B*.
**Figure S10:** Phenotypic analyses of *Tawak3‐b* mutant lines and Fielder in heading stage of Fielder.
**Figure S11:** Grain traits of *Tawak3‐b* mutant lines and Fielder.
**Figure S12:** Phenotypic analyses in heading stage of Fielder and expression of *TaWAK3‐B* in *TaWAK3‐B‐OE* transgenic plants and Fielder.
**Figure S13:** Grain traits of *TaWAK3‐B‐OE* transgenic plants and Fielder.
**Figure S14:** Validation of DEGs identified in the *d14078* in the Fielder and *Tawak3‐b* mutant.
**Figure S15:** Subcellular location of TaADF3‐A‐GFP, TaKLCR1‐A‐GFP and TaIQD2‐D‐GFP in *N. benthamiana* cells.
**Figure S16:** Schematic illustration of full‐length and different lengths of TaWAK3‐B.
**Figure S17:** Physical interaction of TaADF3‐A‐nLUC, TaKLCR1‐A‐nLUC and TaIQD2‐D‐nLUC with TaWAK3‐B^STK^‐cLUC and TaWAK3‐B^GUB^‐cLUC confirmed by the SLC assays.
**Figure S18:** Alignment of TaKLCR1‐A/B/D protein sequence.
**Figure S19:** Amino acid sequence alignment of CRISPR/Cas9‐mediated mutations in the *TaKLCR1‐A/B/D*.
**Figure S20:** Phenotypic analyses of *Taklcr1* mutant lines and Fielder in heading stage of Fielder.
**Figure S21:** Grain traits of *Taklcr1* mutant lines and Fielder.
**Figure S22:** TaWAK3‐B^E938K^ in *d14078* attenuates normal responses to exogenous applications of brassinosteroid (BR) phytohormone.

## Data Availability

The data that supports the findings of this study are available in the Supporting Information of this article.
