# Supplementary material for: A Single‐Base Mutation in TaWAK3‐B Reduces Plant Height via Cytoskeleton in Bread Wheat
Source: Plant Biotechnol J. 2026 Jan 29;24(5):3261–78. doi: 10.1111/pbi.70563 (PMC13110162; doi:10.1111/pbi.70563)
Supplement: Supplementary file 7 — Figure S1: Grain traits of Jinmai47 and d14078. Figure S2: Plant phenotypes and grain traits of Jing411, d14078 and Jinmai47. Figure S3: A major QTL Controlling plant height on 6B chromosome. Figure S4: Genetic linkage map and QTL analysis of QPh.cau‐6B. Figure S5: Transcription levels of candidate genes in the QPh.cau‐6B mapping interval by RNA‐seq. Figure S6: Nucleotide sequence alignment of TaWAK3‐B gene from Jinmai47 and d14078. Figure S7: Amino acid sequence alignment of TaWAK3‐B from Jinmai47 and d14078. Figure S8: Phylogenetic analysis of TaWAK3‐B sequences from different species. Figure S9: Amino acid sequence alignment of CRISPR/Cas9‐mediated mutations in the TaWAK3‐B. Figure S10: Phenotypic analyses of Tawak3‐b mutant lines and Fielder in heading stage of Fielder. Figure S11: Grain traits of Tawak3‐b mutant lines and Fielder. Figure S12: Phenotypic analyses in heading stage of Fielder and expression of TaWAK3‐B in TaWAK3‐B‐OE transgenic plants and Fielder. Figure S13: Grain traits of TaWAK3‐B‐OE transgenic plants and Fielder. Figure S14: Validation of DEGs identified in the d14078 in the Fielder and Tawak3‐b mutant. Figure S15: Subcellular location of TaADF3‐A‐GFP, TaKLCR1‐A‐GFP and TaIQD2‐D‐GFP in N. benthamiana cells. Figure S16: Schematic illustration of full‐length and different lengths of TaWAK3‐B. Figure S17: Physical interaction of TaADF3‐A‐nLUC, TaKLCR1‐A‐nLUC and TaIQD2‐D‐nLUC with TaWAK3‐BSTK‐cLUC and TaWAK3‐BGUB‐cLUC confirmed by the SLC assays. Figure S18: Alignment of TaKLCR1‐A/B/D protein sequence. Figure S19: Amino acid sequence alignment of CRISPR/Cas9‐mediated mutations in the TaKLCR1‐A/B/D. Figure S20: Phenotypic analyses of Taklcr1 mutant lines and Fielder in heading stage of Fielder. Figure S21: Grain traits of Taklcr1 mutant lines and Fielder. Figure S22: TaWAK3‐BE938K in d14078 attenuates normal responses to exogenous applications of brassinosteroid (BR) phytohormone. [file PBI-24-3261-s005.docx]

**SUPPORTING INFORMATION:**

**
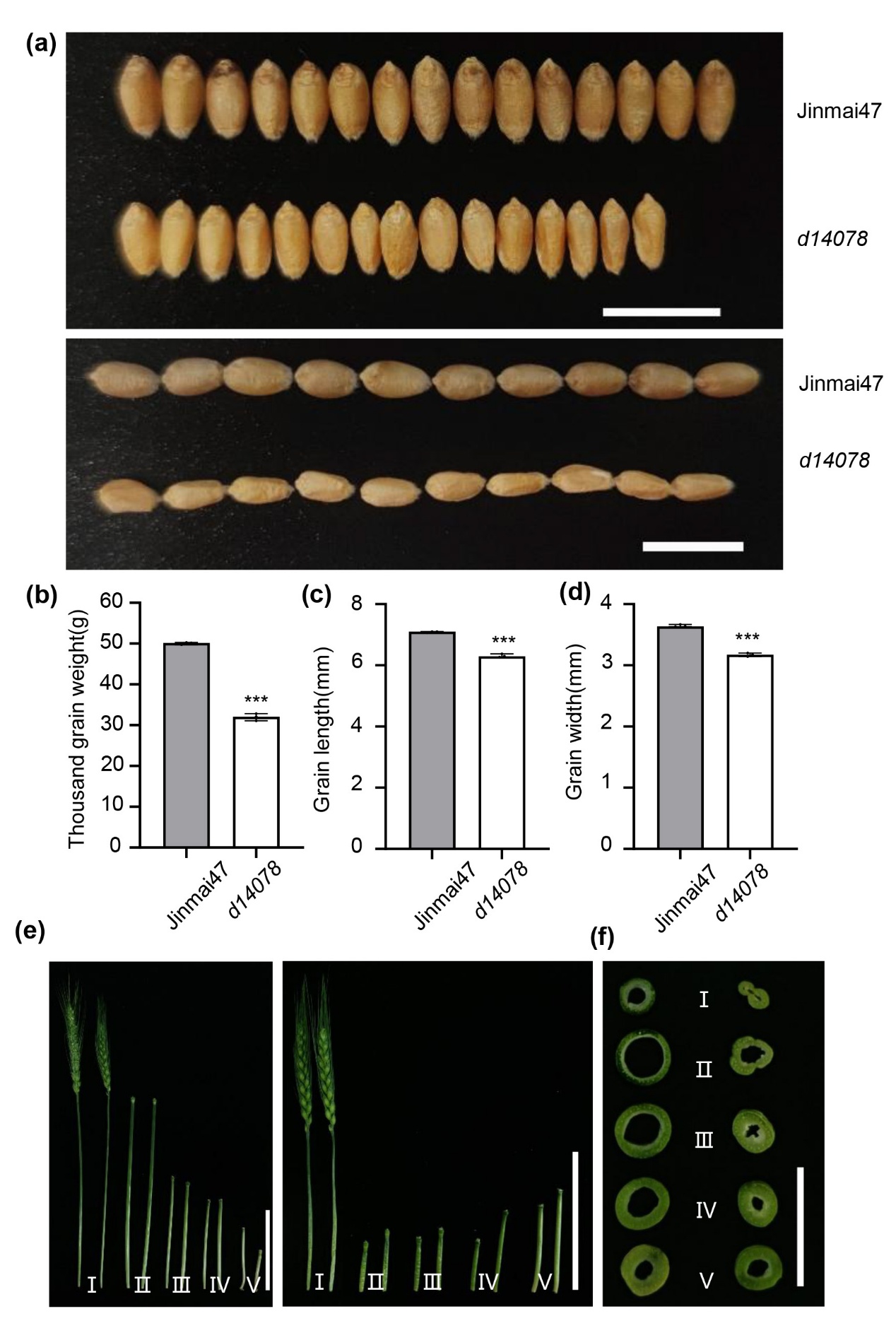
**

**Figure S1 Grain traits of Jinmai47 and *d14078*.** (a) Grain traits of Jinmai47 and *d14078*, bars, 1 cm. (b-d) Statistical analyses of thousand grain weight (b), grain length (c) and grain width (d) of Jinmai47 and *d14078* (*n* = 3). (e) The stem segments of *d14078* mutant plants were wrinkled and smaller. Bars, 10 cm. Ⅰ-IⅤ indicate the different stem segments of Jinmai47 and *d14078*. (f) Cross section of different segments from the Jinmai47 and *d14078*. Bars, 1 cm. SD, standard deviation. (Student’s *t*-test ***, *P* < 0.001).


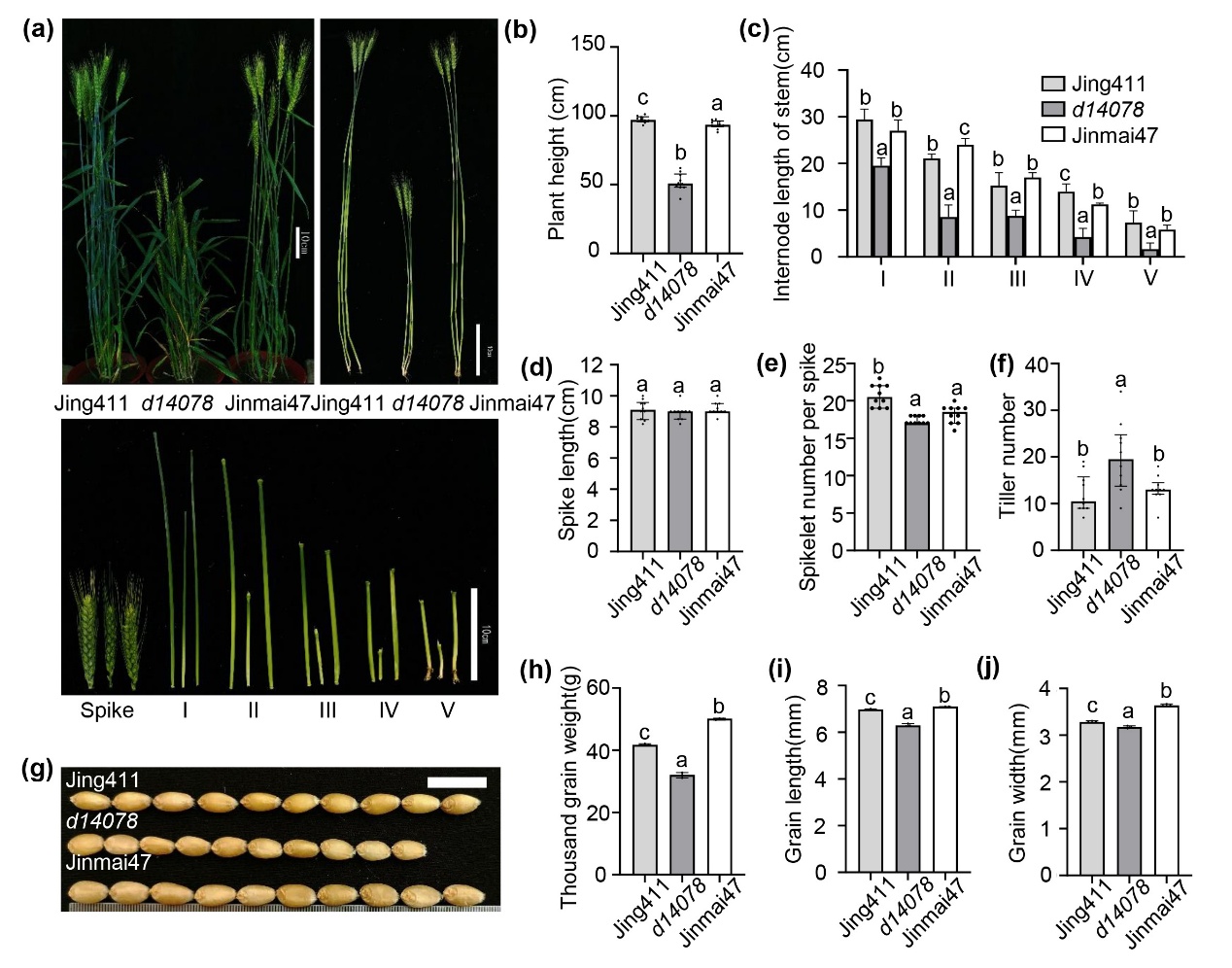


**Figure S2** **Plant phenotypes and** **grain traits of Jing411, *d14078* and Jinmai47.** (a) Plant height of Jing411, *d14078* and Jinmai47, bars, 5 cm. (b-f) Statistical analyses of plant height (b), internode length of stems (c), spike length (d), spikelet number per spike (e) and tiller number (f) of Jing411, *d14078* and Jinmai47(*n* = 10). (g) Grain traits of Jing411, *d14078* and Jinmai47, bars, 1 cm. (h-j) Statistical analyses of thousand grain weight (h), grain length (i) and grain width (j) of Jing411, *d14078* and Jinmai47(*n* = 3). Data are means ± SD. SD, standard deviation. The different letters above the bars show significant differences [one way ANOVA, Tukey’s post hoc test, *P* < 0.05].


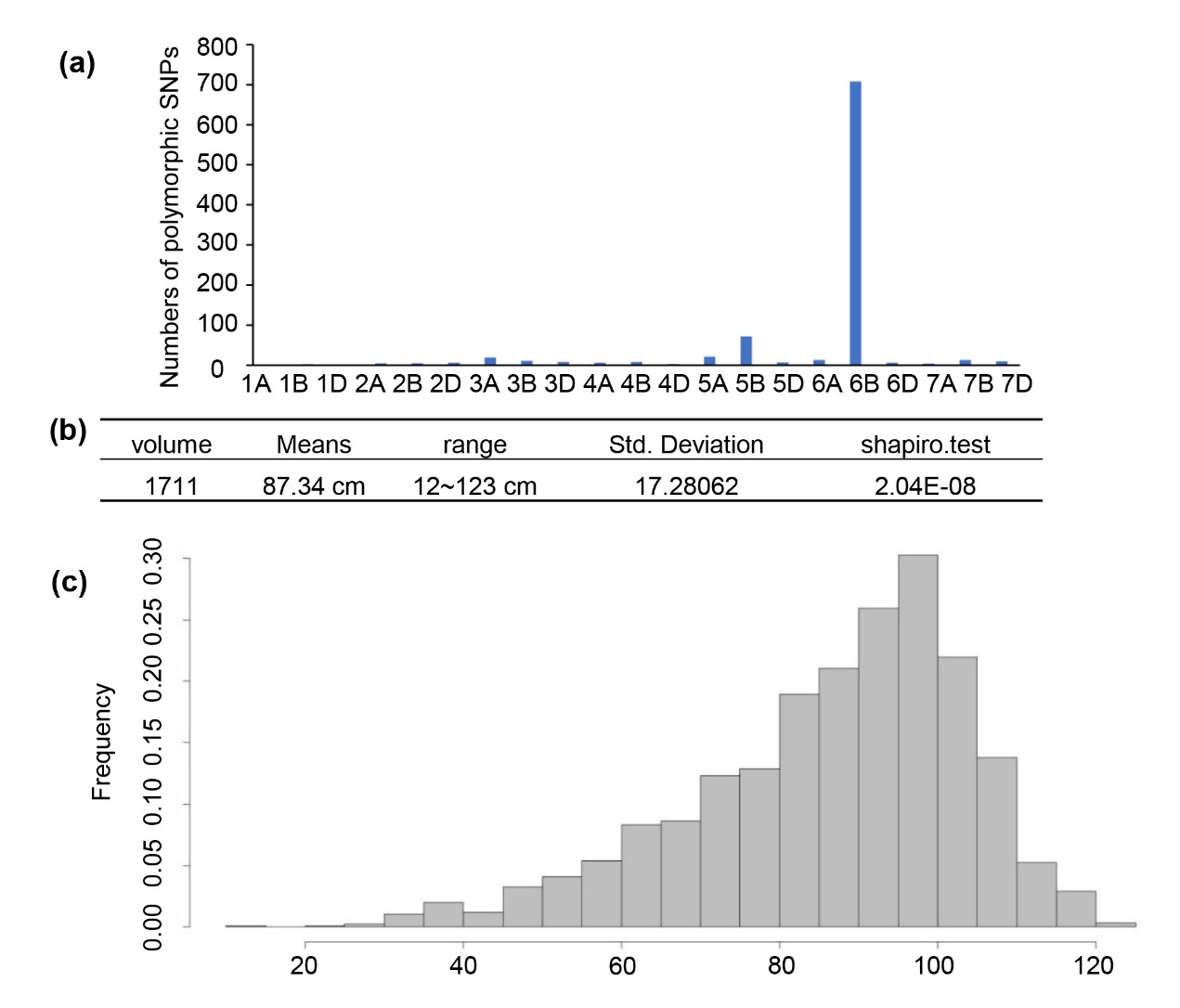


**Figure S3 A major QTL Controlling plant height on 6B chromosome.**

(a) Enrichment analysis of single-nucleotide polymorphisms (SNPs) based Bulked Segregant Analysis (BSA). See also Table S2. (b) The plant height analysis of F_2_ separated population of Jing411 and *d14078.* (c) Histogram of plant height frequency distribution of F_2_ separated population of Jing411 and *d14078*.


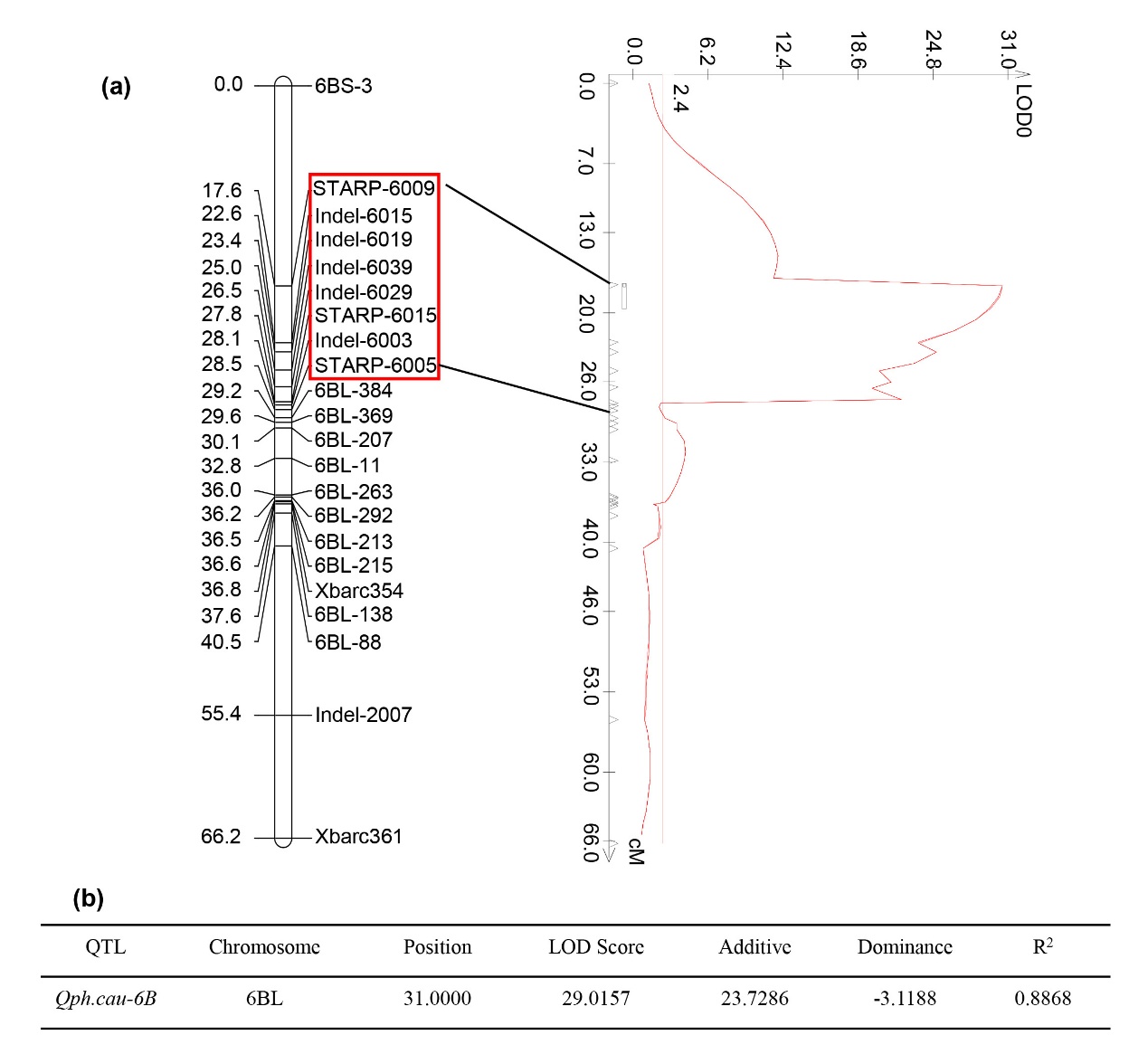


**Figure S4 Genetic linkage map and QTL analysis of *QPh.cau-6B.*** (a) Genetic linkage map analysis of the *QPh.cau-6B*. (b) The chromosome, position, LOD score, additive and dominance of *QPh.cau-6B.*


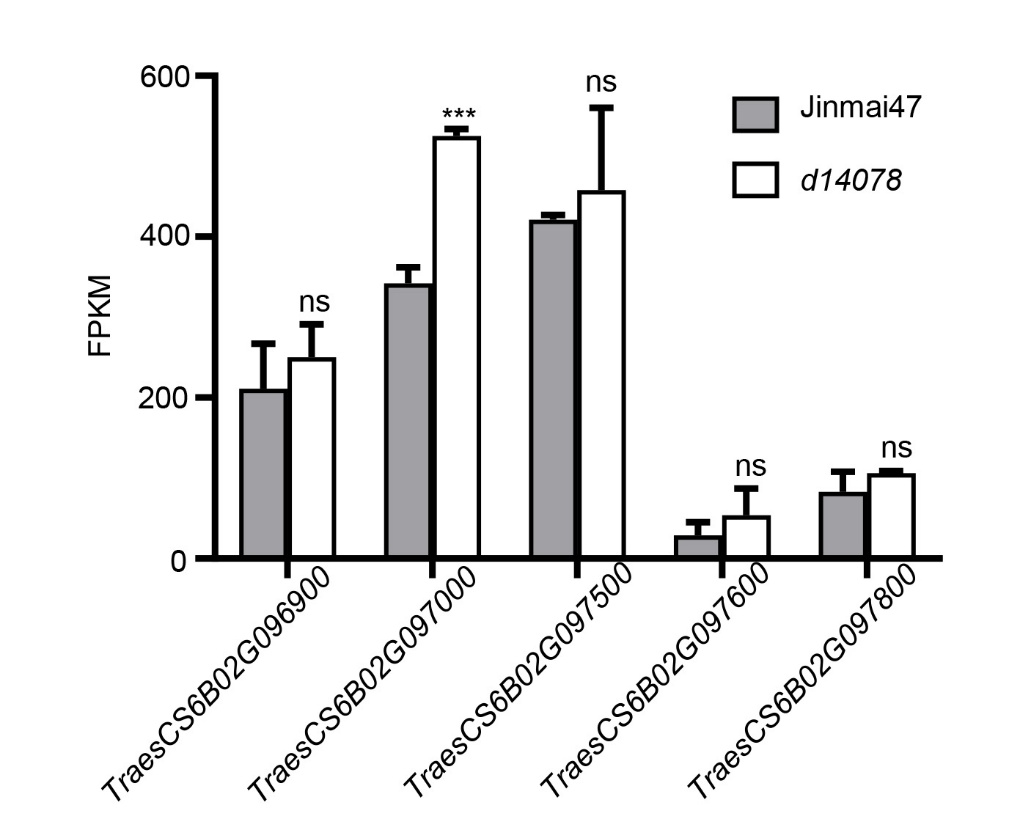


**Figure S5 Transcription levels of candidate genes in the *QPh.cau-6B* mapping interval by RNA-seq.** FPKM, fragments per kilobase of exon model per million mapped fragments. Data are means ± SD (*n* = 3). (Student’s *t*-test, ***, *P* < 0.001; ns, not significant).


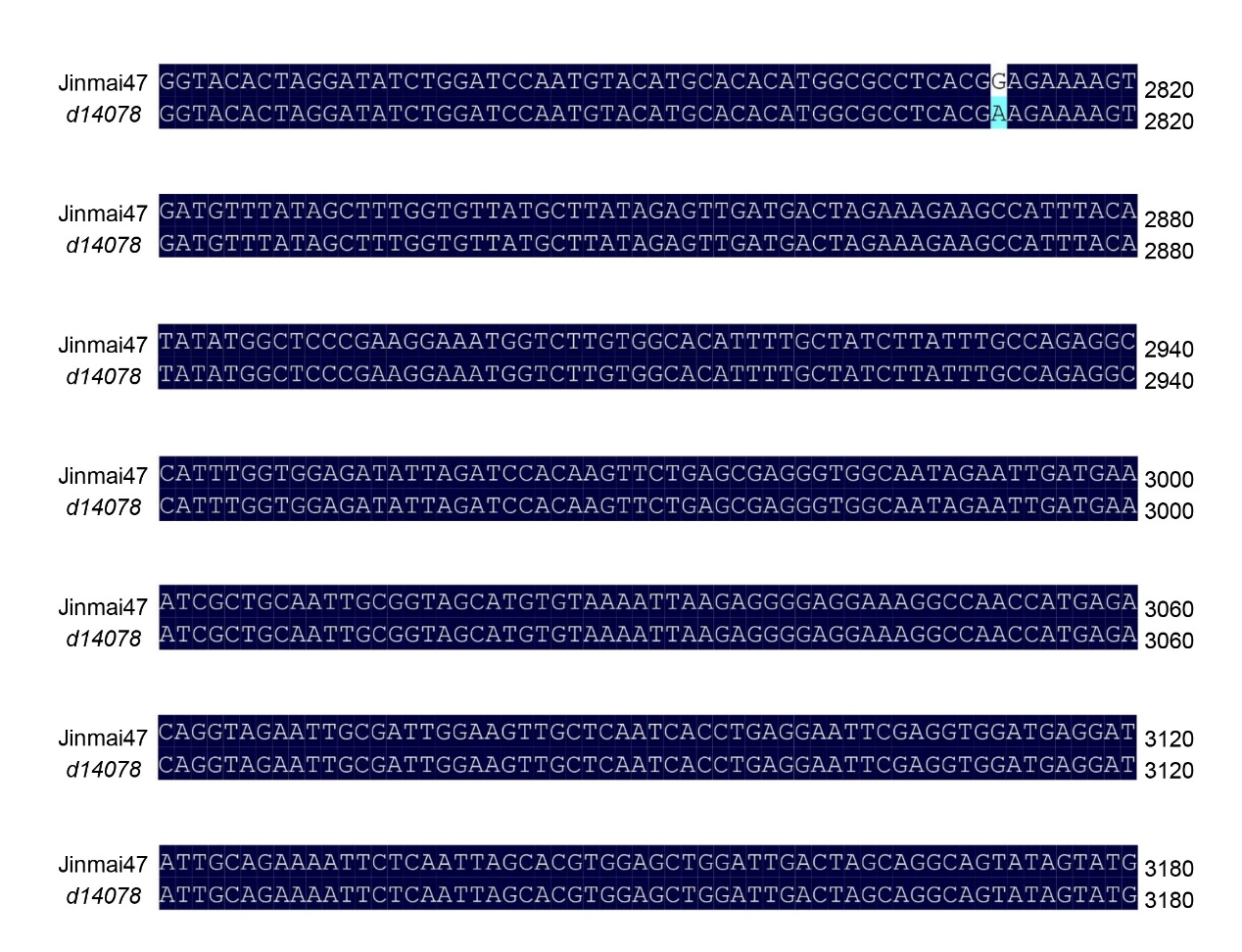


**Figure S6 Nucleotide sequence alignment of *TaWAK3-B* gene from Jinmai47 and *d14078*.**


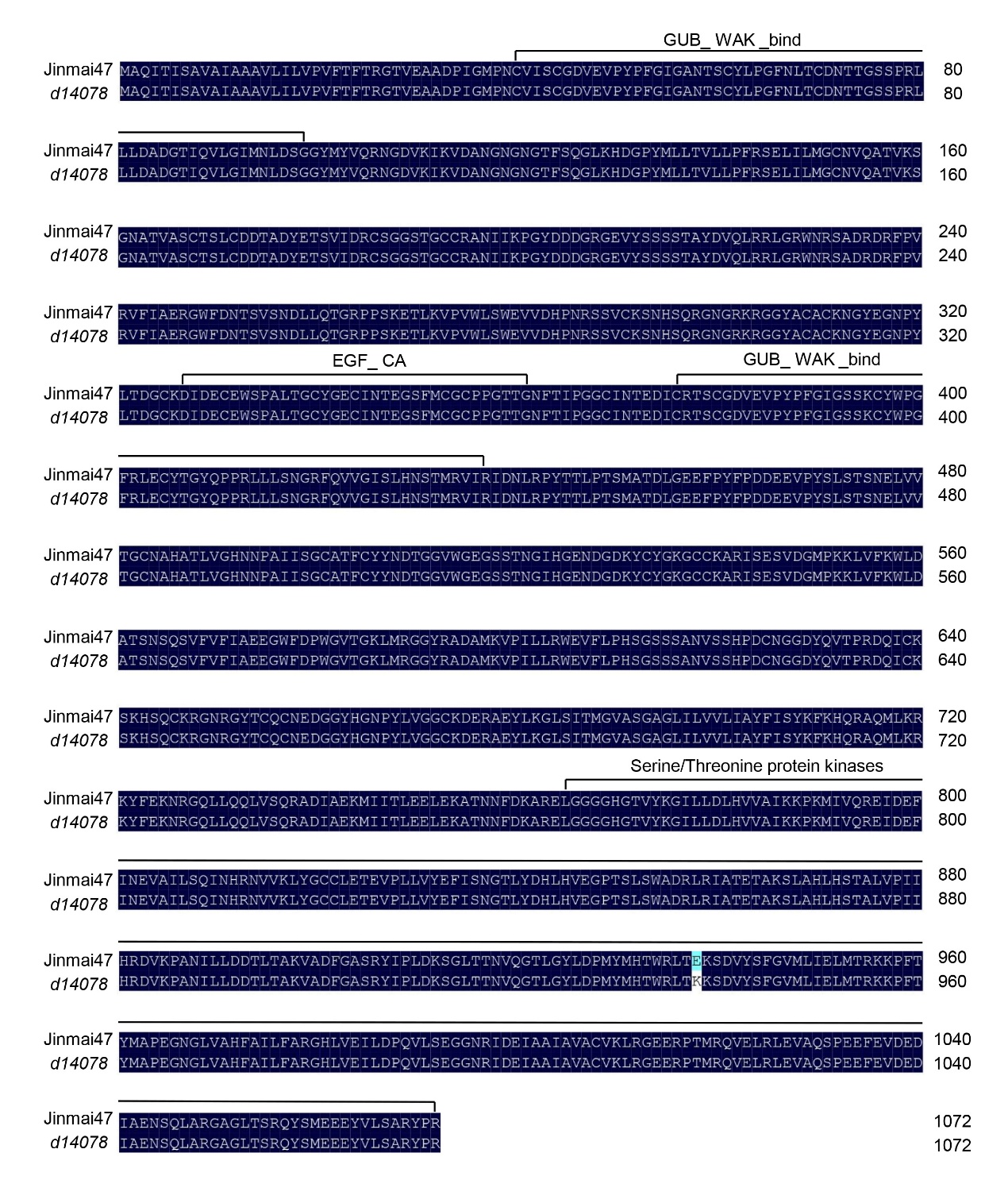


**Figure S7 Amino acid sequence alignment of TaWAK3-B from Jinmai47 and *d14078*.** GUB_WAK, EGF_CA and Serine/threonine protein kinase indicate the domain of TaWAK3-B.


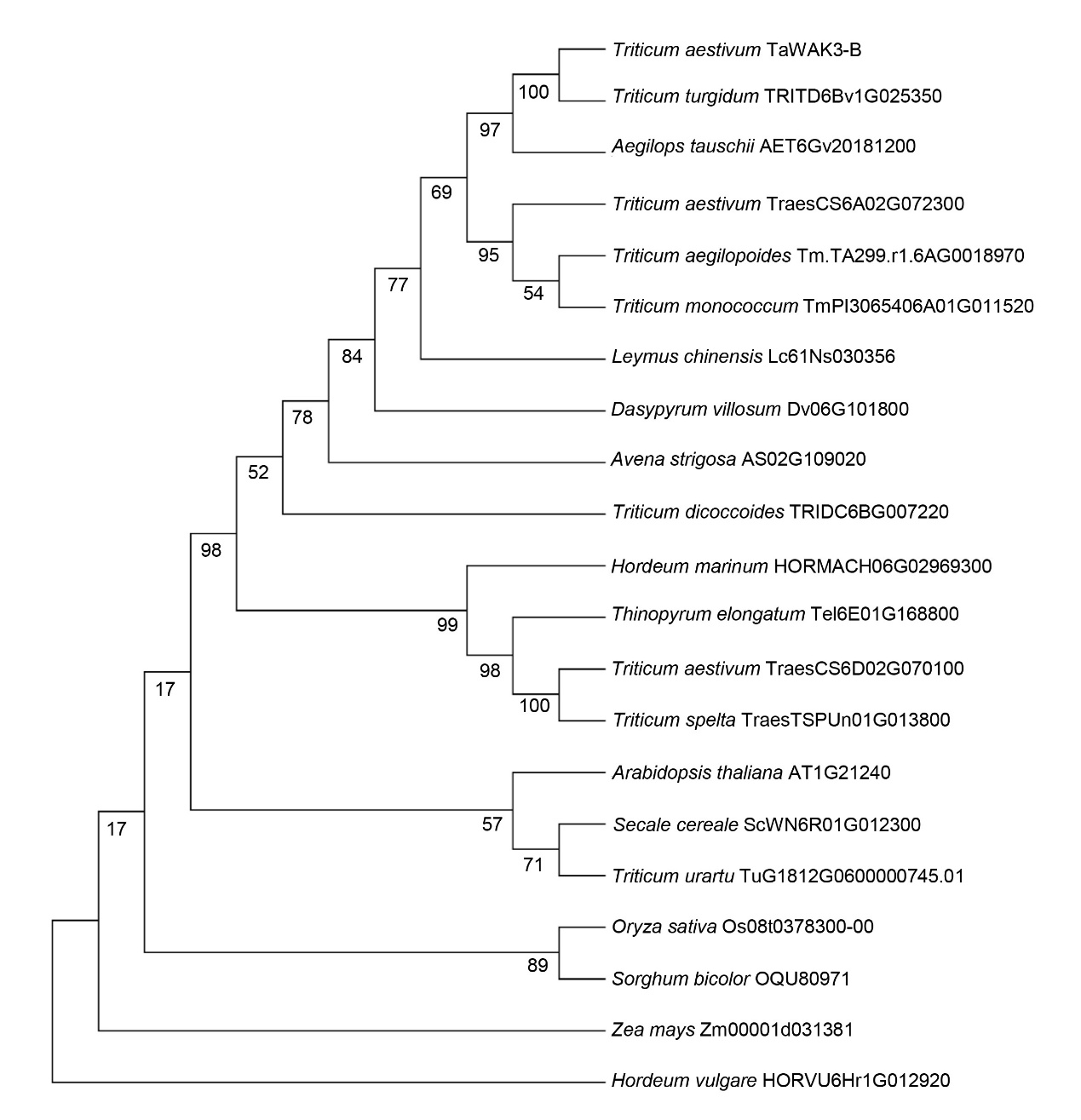


**Figure S8 Phylogenetic analysis of TaWAK3-B sequences from different species.**


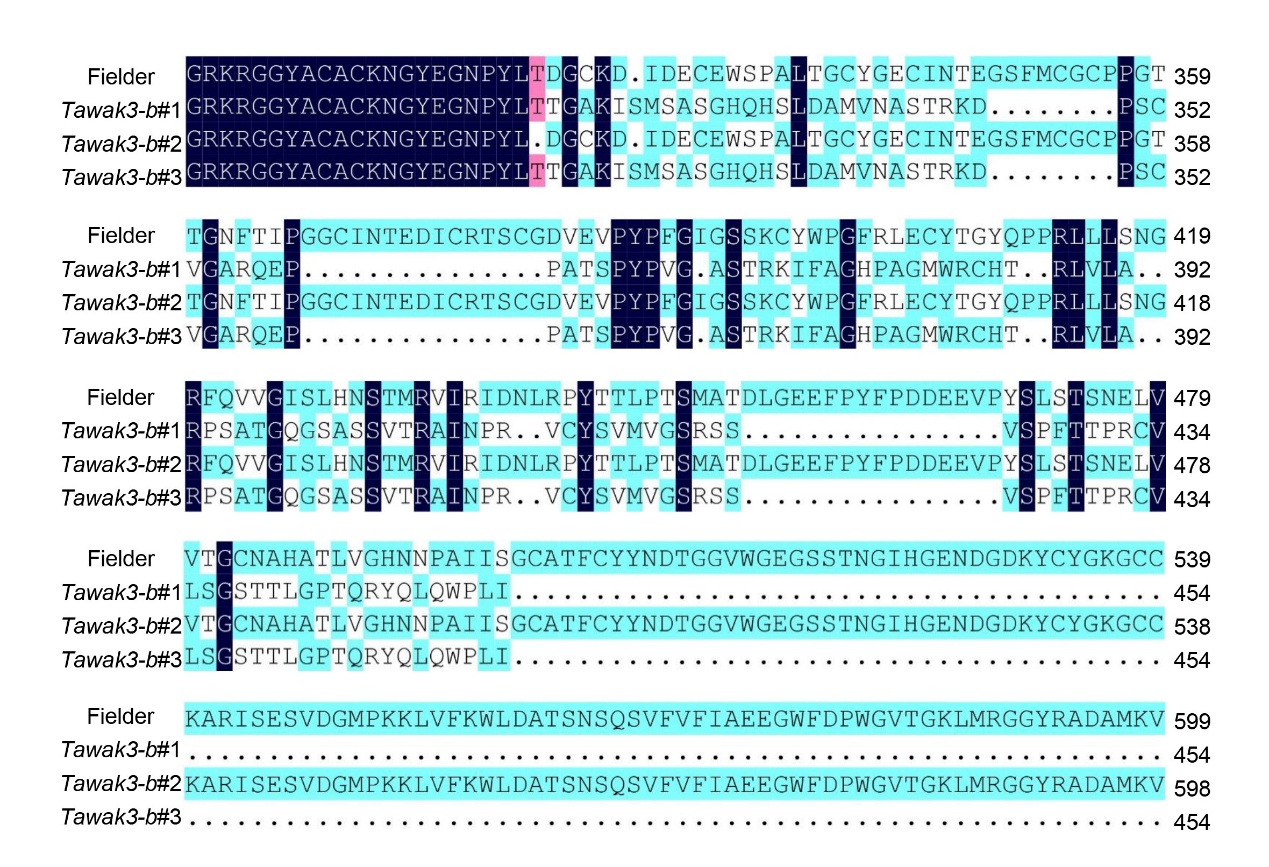


**Figure S9 Amino acid sequence alignment of CRISPR/Cas9-mediated mutations in the *TaWAK3-B*.**


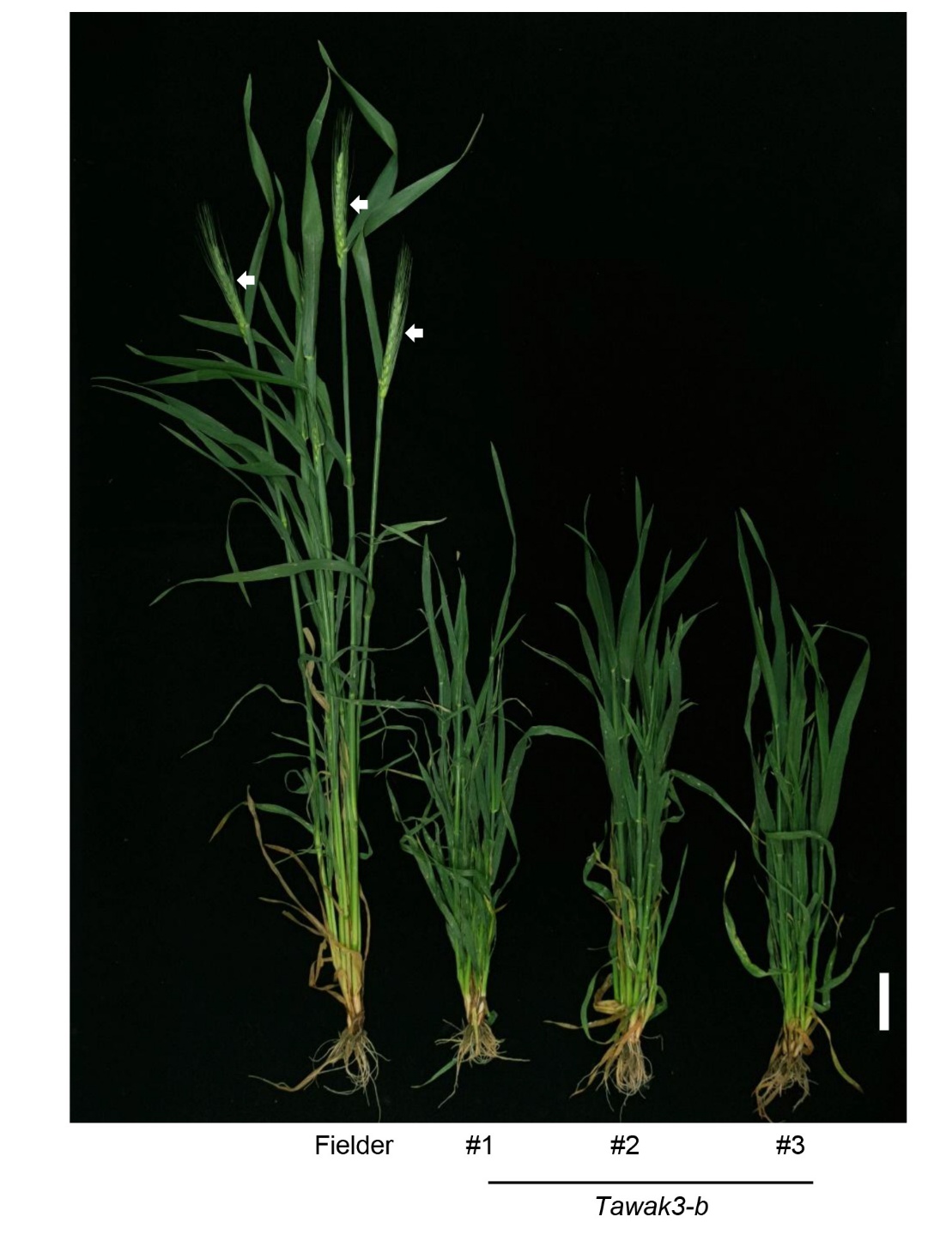


**Figure S10 Phenotypic analyses of *Tawak3-b* mutant lines and Fielder in heading stage of Fielder.** The white arrows indicate the spikes of Fielder. Bar, 5 cm.


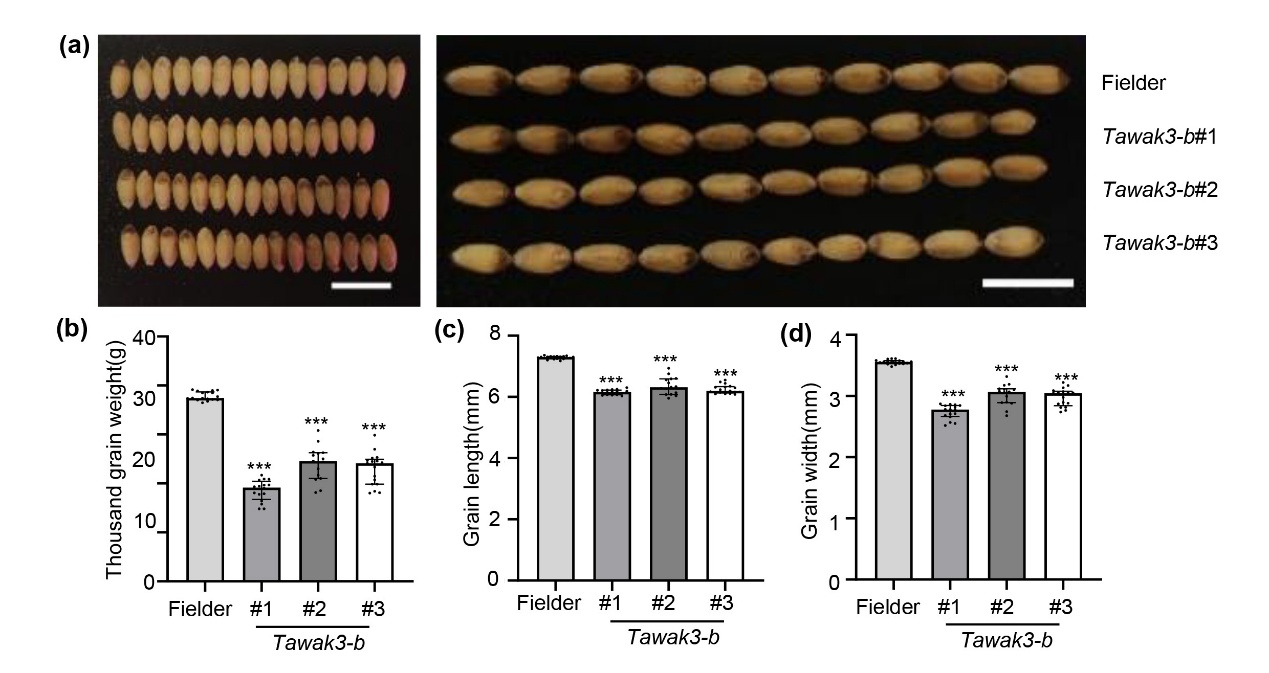


**Figure S11 Grain traits of *Tawak3-b* mutant lines and Fielder.** (a) Grain traits of Fielder and *Tawak3-b* mutant. Bars, 1 cm. (b-d) Statistical analysis of thousand grain weight (b), grain length (c) and grain width (d) of Fielder and *Tawak3-b* mutant (*n*^Fielder^= 18, *n^Tawak3-b^*^#1^= 16, *n^Tawak3-b^*^#2^ = 15 and *n^Tawak3-b^*^#3^ = 17). Data are means ± SD. (Student’s *t*-test, ***, *P* < 0.001).


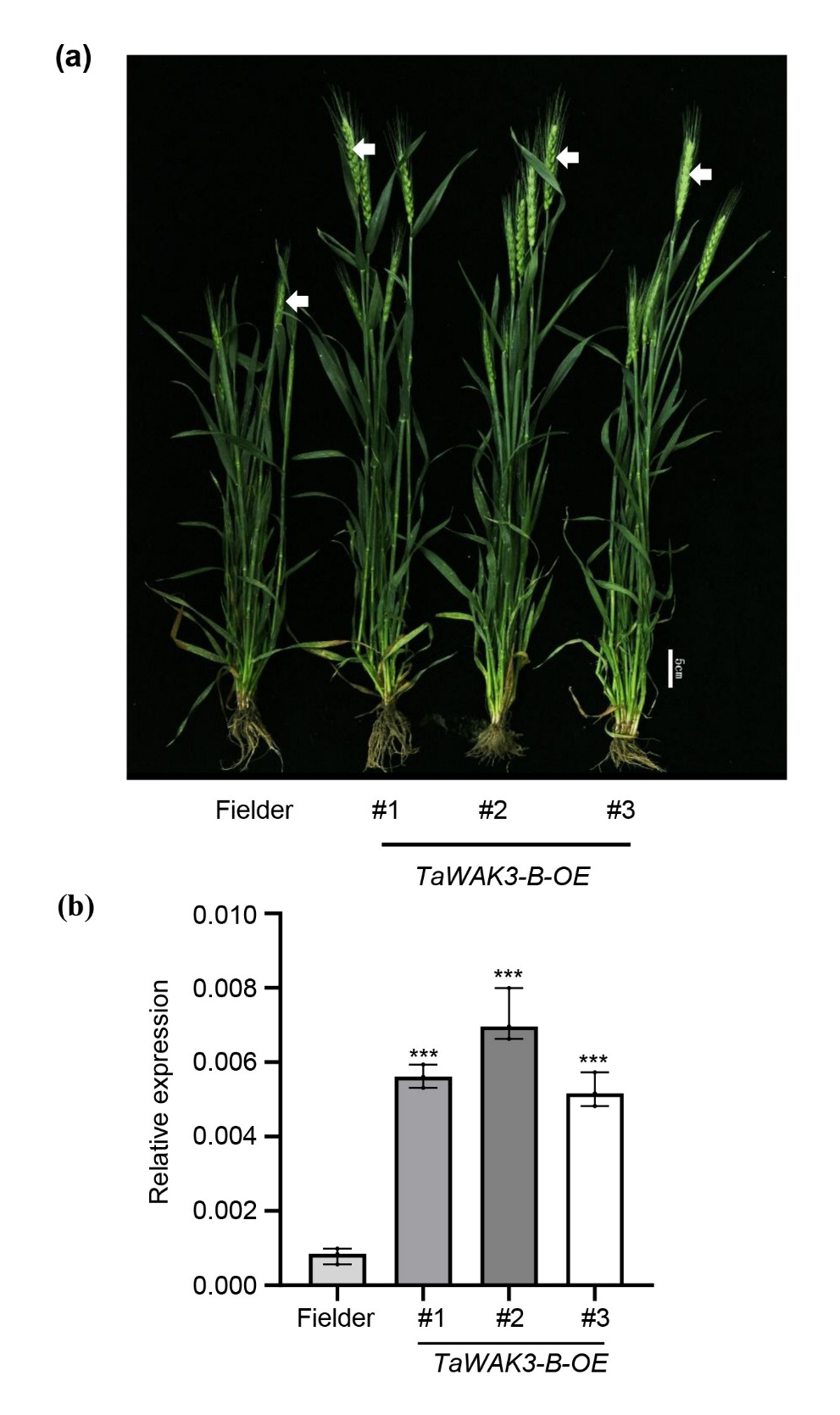


**Figure S12 Phenotypic analyses in heading stage of Fielder and expression of *TaWAK3-B* in *TaWAK3-B-OE* transgenic plants and Fielder.** (a) Phenotypic analyses of *TaWAK3-B-OE* mutants and Fielder. Bar, 10 cm. (b) RT-qPCR showing the expression levels of *TaWAK3-B* in *TaWAK3-B-OE* transgenic plants and Fielder. Data are means ± SD (*n* = 3), (Student’s *t*-test, ***, *P* < 0.001).


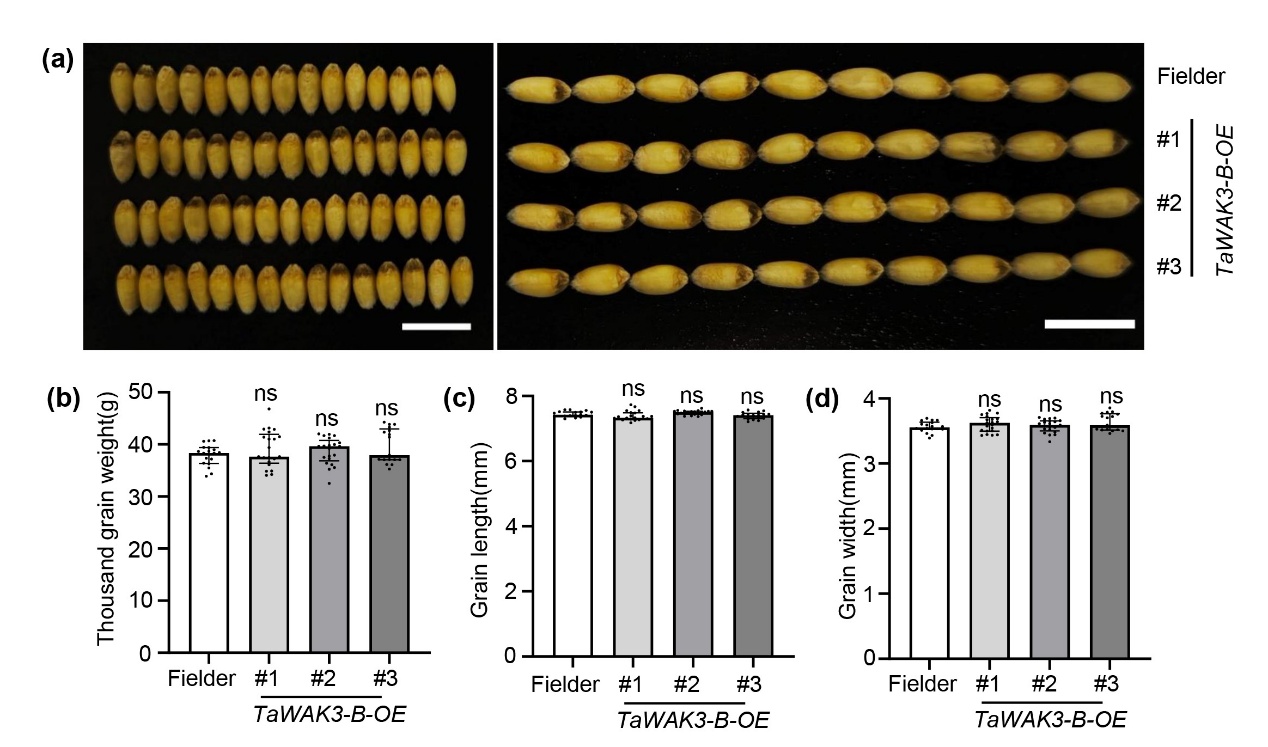


**Figure S13 Grain traits of *TaWAK3-B-OE* transgenic plants and Fielder.** (a) Grain traits of Fielder and *TaWAK3-B-OE* transgenic plants. Bars, 1 cm. (b-d) Statistical analysis of thousand grain weight (b), grain length (c) and grain width (d) of Fielder and *TaWAK3-B-OE* transgenic plants (*n*^Fielder^= 18, *n^TAWAK3-B-OE^*^#1^= 21, *n^TAWAK3-B-OE^*^#2^ = 21 and *n^TAWAK3-B-OE^*^#3^ = 17). Data are means ± SD. (Student’s *t*-test, ns, not significant).


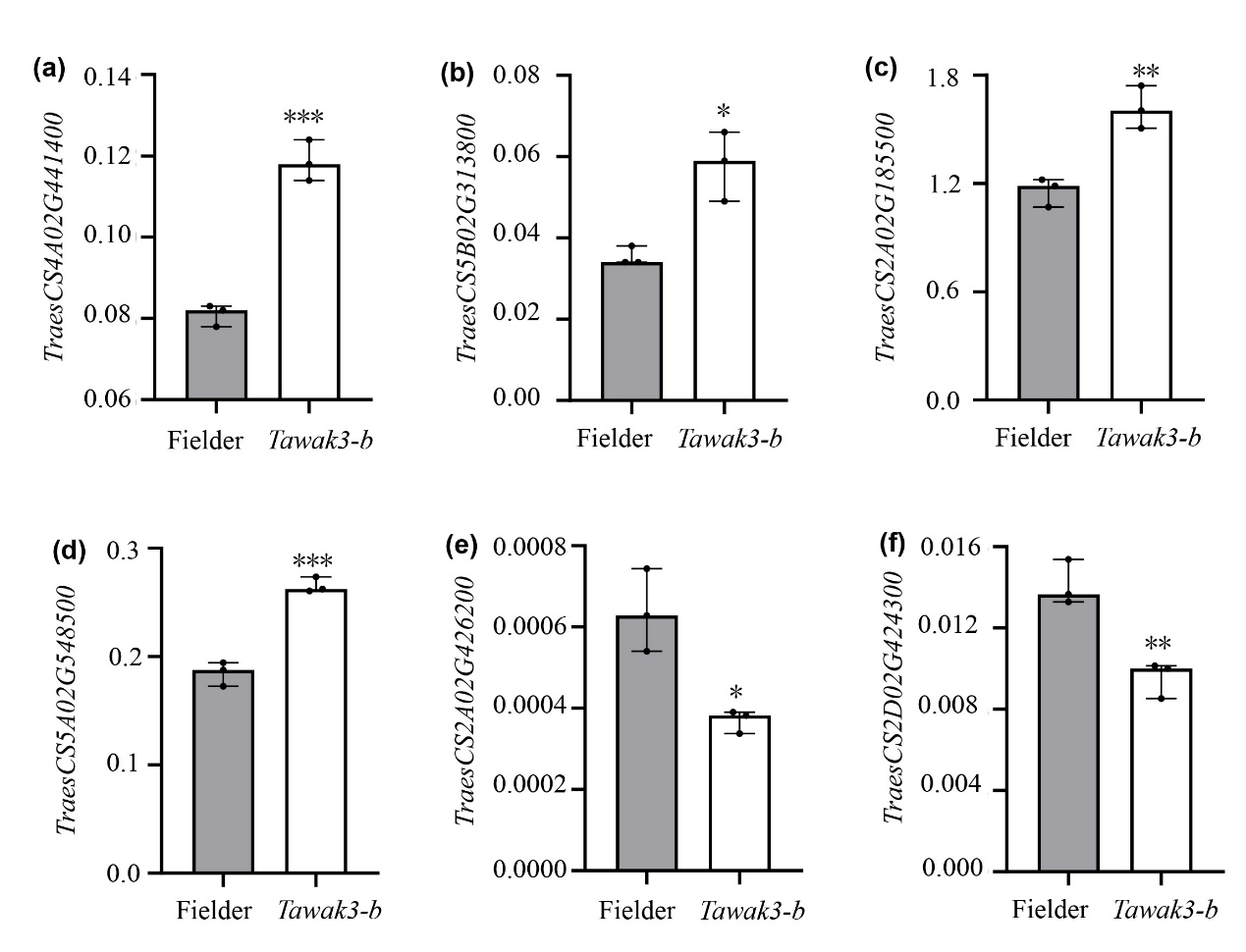


**Figure S14 Validation of DEGs identified in the *d14078* in the Fielder and *Tawak3-b* mutant.** (a-b) *TraesCS4A02G441400* and *TraesCS5B02G313800* are negative regulation of organ growth genes (*n*=3). (c) *TraesCS2A02G185500* is microtubule-based process gene (*n*=3). (d) *TraesCS5A02G548500* is cell wall macromolecule catabolic process gene (*n*=3). (e-f) *TraesCS2A02G426200* and *TraesCS2D02G424300* are regulation of stem cell division genes (*n*=3). Data are means ± SD. SD, standard deviation. (Student’s *t*-test, *, *P* < 0.05, **, *P* < 0.01, ***, *P* < 0.001).


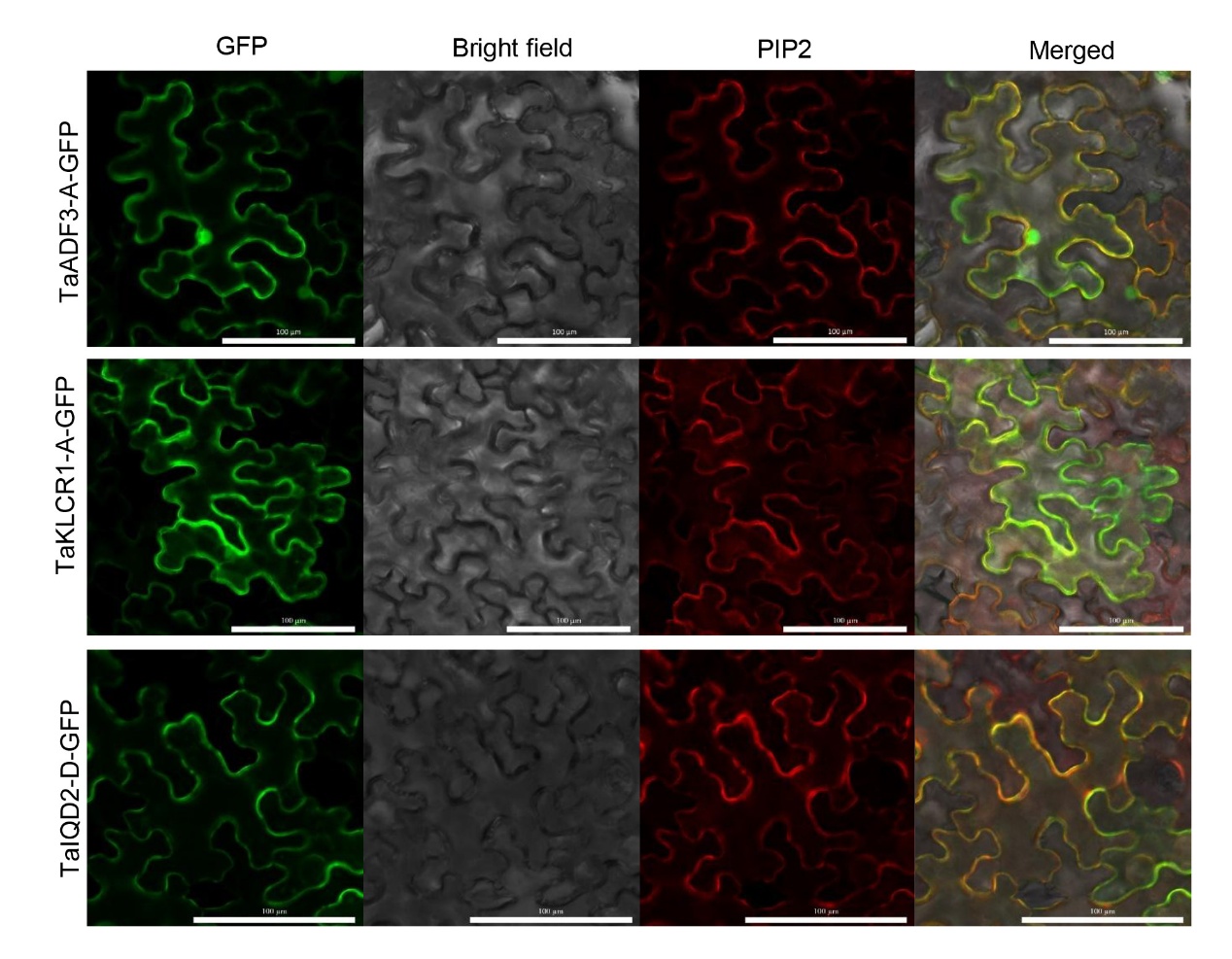


**Figure S15 Subcellular location of TaADF3-A-GFP, TaKLCR1-A-GFP and TaIQD2-D-GFP in *N. benthamiana* cells.** PIP2 is used as a marker of cell membrane. Bars, 100μm.


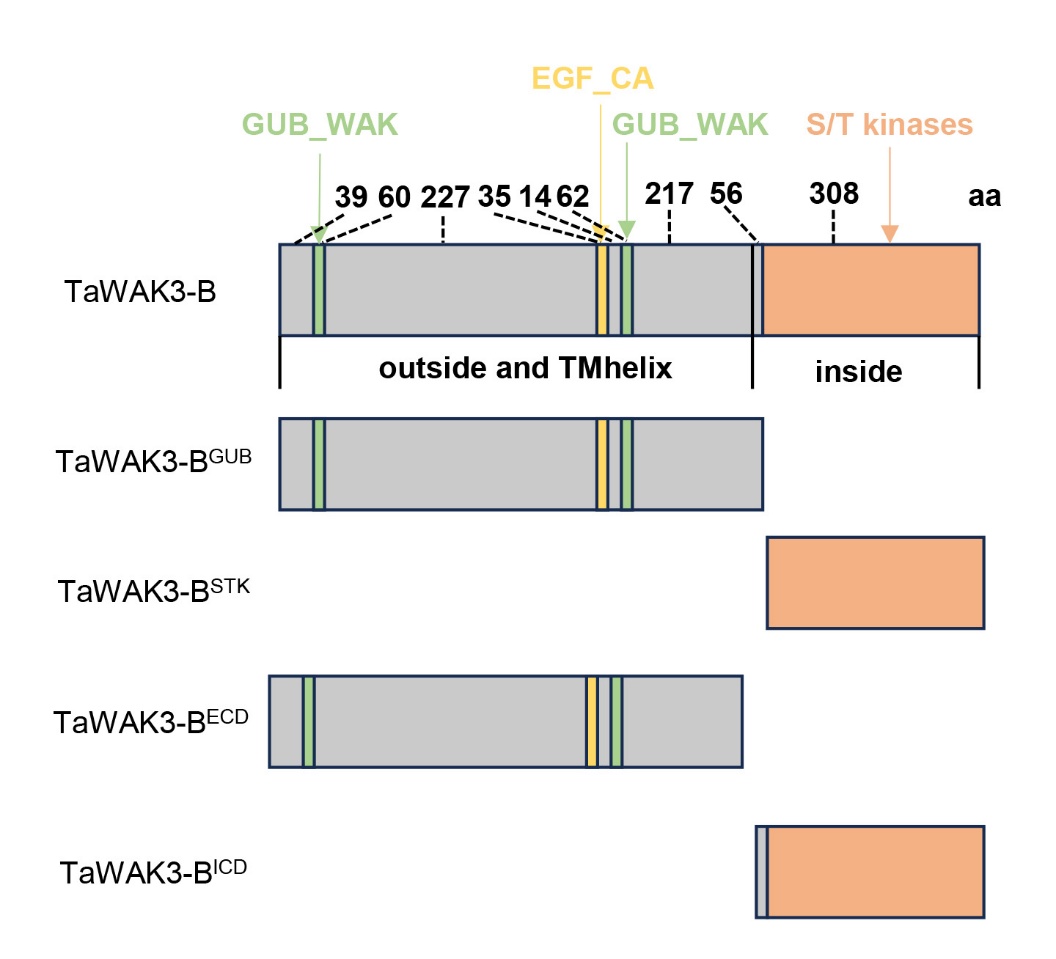


**Figure S16 Schematic illustration of full-length and different lengths of TaWAK3-B.** TaWAK3-B, the full-length protein of TaWAK3-B; TaWAK3-B^GUB^, the wall-associated receptor kinase galacturonan-binding (GUB) and calcium-binding EGF-like (EGF_CA) domain of TaWAK3-B; TaWAK3-B^STK^, the kinase domain (S/T kinases) of TaWAK3-B; TaWAK3-B^ECD^, the transmembrane domain of TaWAK3-B; TaWAK3-B^ICD^, the intracellular domain of TaWAK3-B.


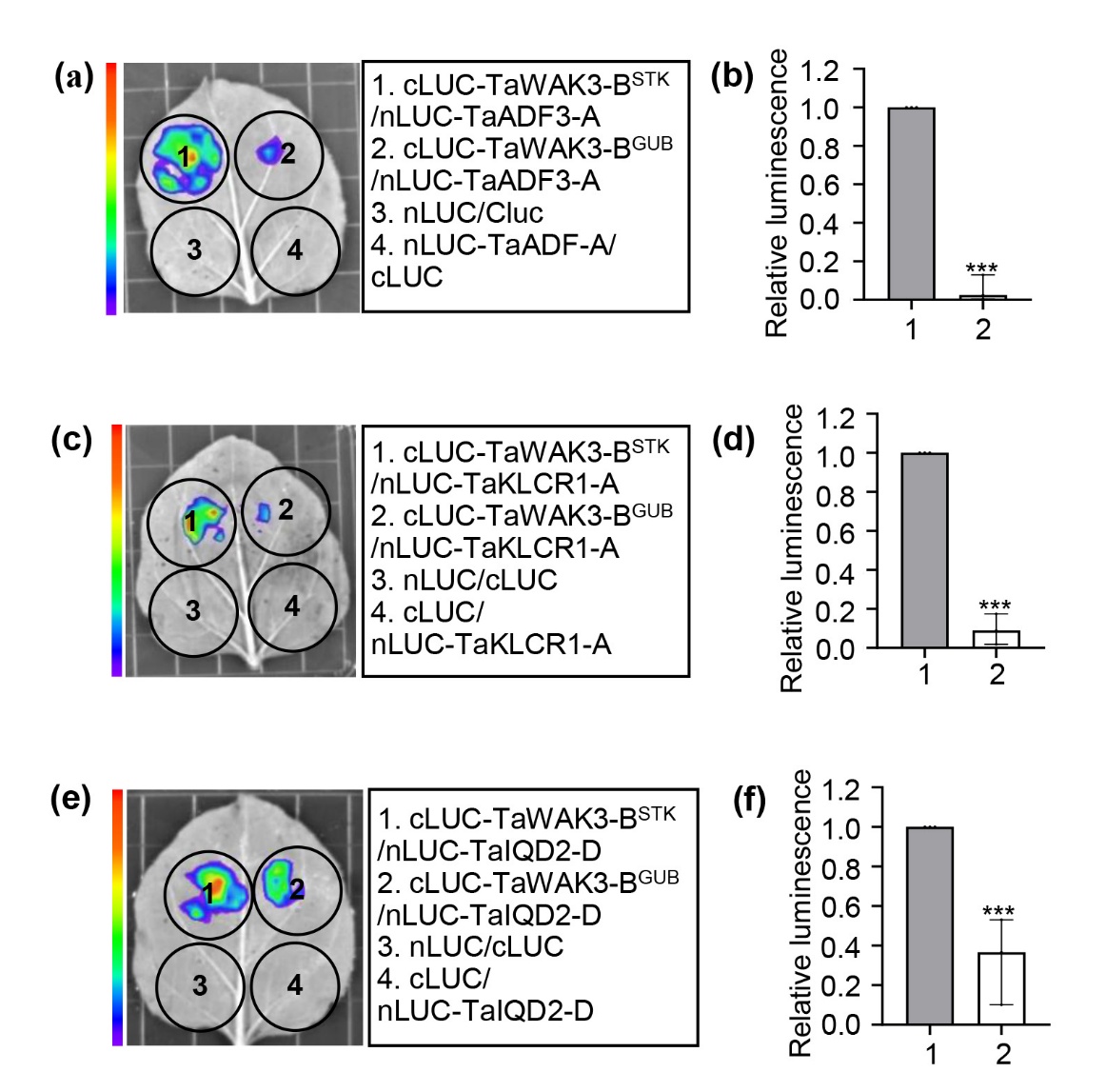


**Figure S17 Physical interaction of TaADF3-A-nLUC, TaKLCR1-A-nLUC and TaIQD2-D-nLUC with TaWAK3-B^STK^-cLUC and TaWAK3-B^GUB^-cLUC confirmed by the SLC assays.** (a) Physical interaction of TaADF3-A-cLUC with TaWAK3-B^STK^-nLUC or TaWAK3-B^GUB^-nLUC confirmed by a SLC assay. (b) Statistic of fluorescence value in (a), and data are means ± SD (*n*=3). (c) Physical interaction of TaKLCR1-A-cLUC with TaWAK3-B^STK^-nLUC or TaWAK3-B^GUB^-nLUC confirmed by a SLC assay. (d) Statistic of fluorescence value in (c), and data are means ± SD (*n*=3). (e) Physical interaction of TaIQD2-D-cLUC with TaWAK3-B^STK^-nLUC or TaWAK3-B^GUB^-nLUC confirmed by a SLC assay. (f) Statistic of fluorescence value in (e), data are means ± SD (*n*=3), (Student’s *t*-test, ***, *P* < 0.001).


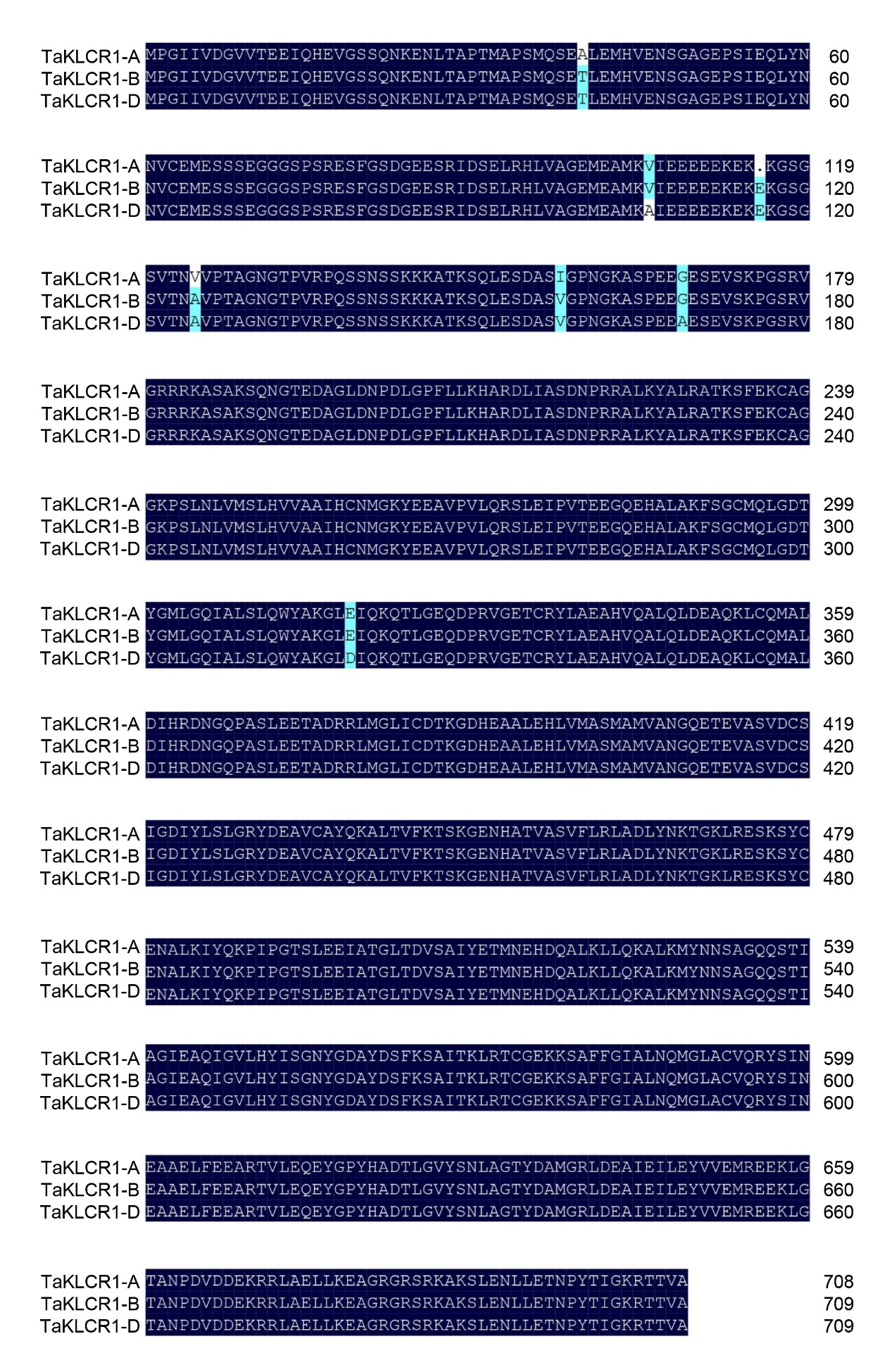


**Figure S18 Alignment of TaKLCR1-A/B/D protein sequences.**


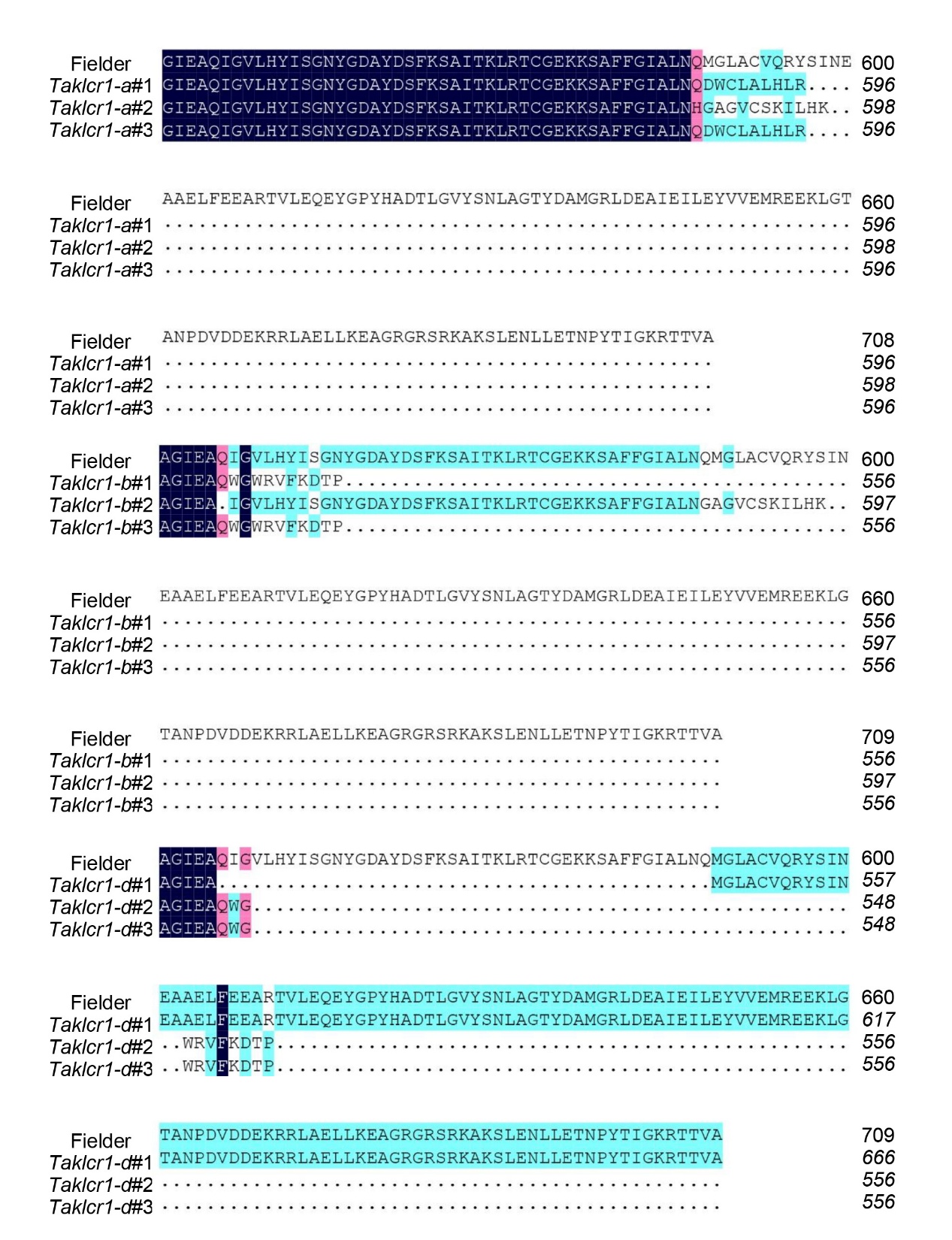


**Figure S19 Amino acid sequences alignment of CRISPR/Cas9-mediated mutations in the *TaKLCR1-A/B/D*.**


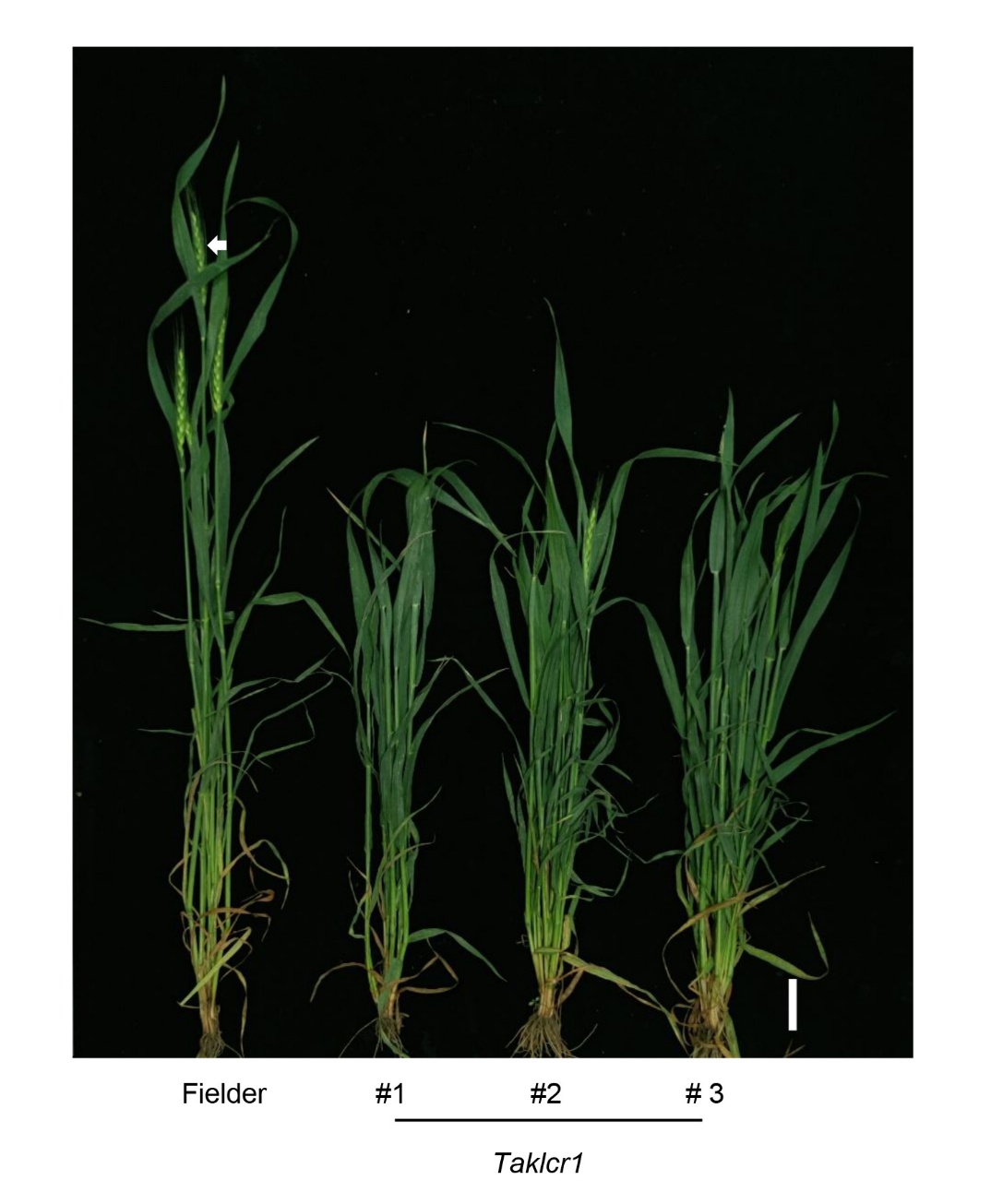


**Figure S20 Phenotypic analyses of *Taklcr1* mutant lines and Fielder in heading stage of Fielder.** The white arrow indicated the spike of Fielder. Bar, 5 cm.


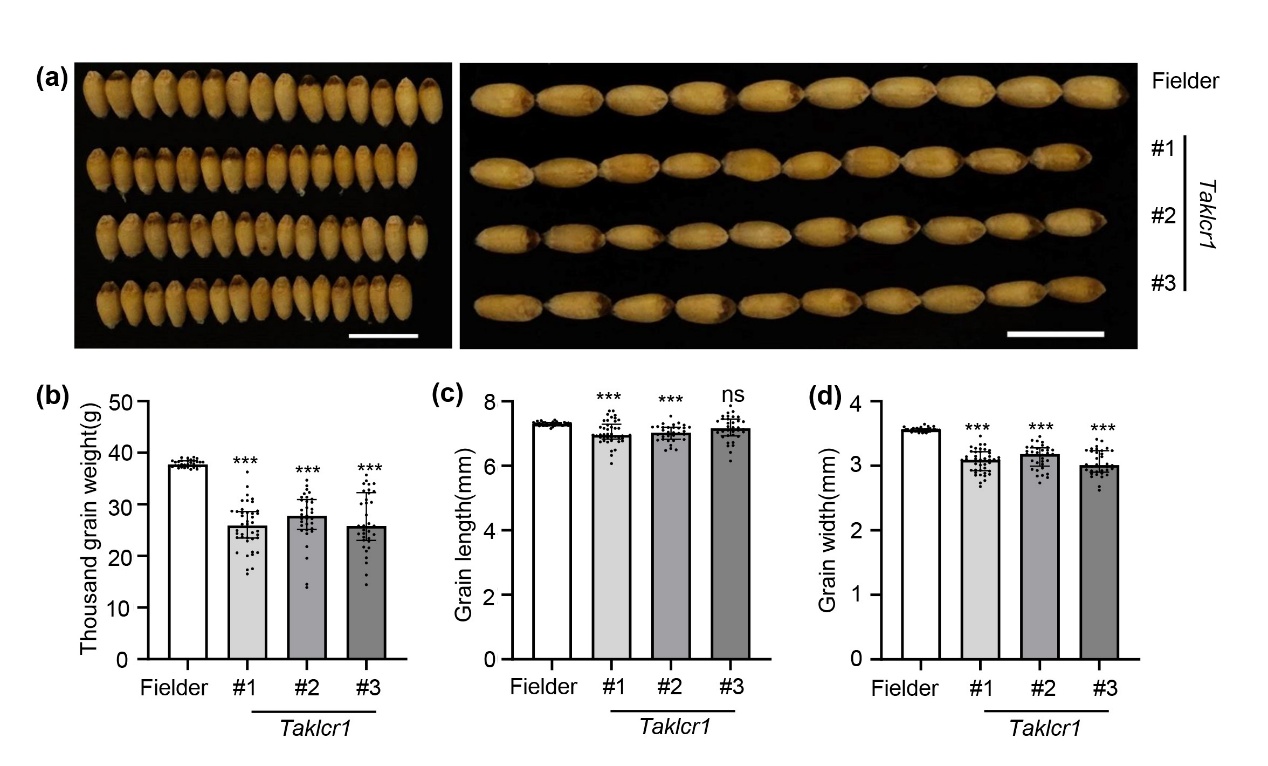


**Figure S21 Grain traits of *Taklcr1* mutant lines and Fielder.** (a) Phenotype of grains of Fielder and *Taklcr1* mutant. Bars, 1 cm. (b-d) Statistical analysis of thousand grain weight (b), grain length (c) and grain width (d) of Fielder and *taklcr1* mutants (*n* ^Fielder^= 34, *n* *^Taklcr1^*^#1^= 42, *n ^Taklcr1^*^#2^ = 33 and *n ^Taklcr1^*^#3^ = 34). Data are means ± SD, (Student’s *t*-test, ns, not significant, ***, *P* < 0.001).


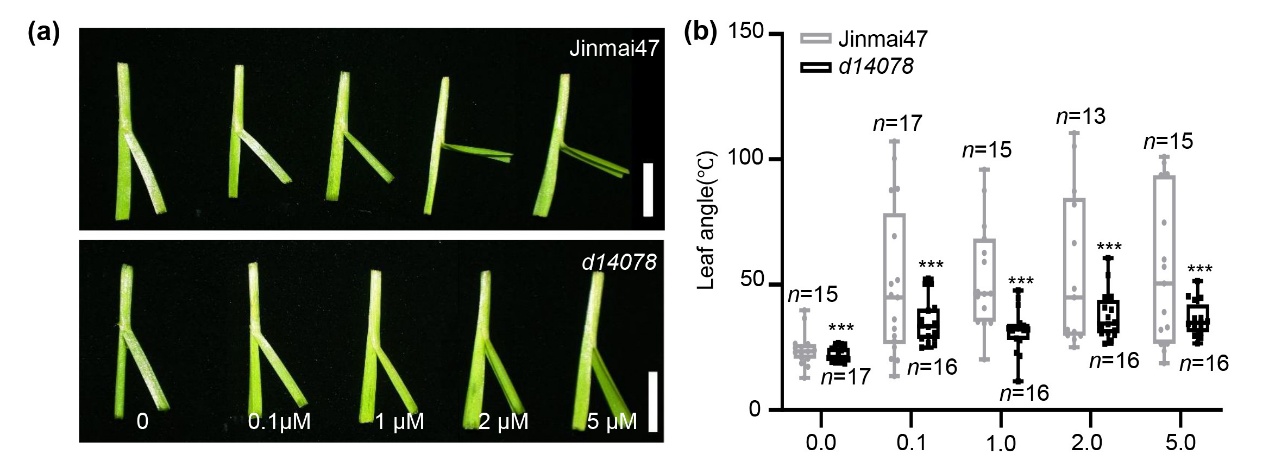


**Figure S22** **TaWAK3-B^E938K^ in *d14078* attenuates normal responses to exogenous applications of brassinosteroid (BR) phytohormone.** (a) Sensitivity of Jinmai47 and *d14078* to BR, represented by the changes in lamina joint inclination. Bars, 1 cm. (b) Statistical analysis of the leaf angle of the seedlings of Jinmai47 and *d14078*. The leaf angles were analyzed by ImageJ software. (Student’s *t*-test, ***, *P* < 0.001).
